# Dynamic Classification of *Plasmodium vivax* Malaria Recurrence: An Application of Classifying Unknown Cause of Failure in Competing Risks

**DOI:** 10.6339/21-jds1026

**Published:** 2021-12-09

**Authors:** Yutong Liu, Feng-Chang Lin, Jessica T. Lin, Quefeng Li

**Affiliations:** 1 Department of Biostatistics, University of North Carolina at Chapel Hill, Chapel Hill, North Carolina, U.S.A.; 2 Institute of Global Health and Infectious Diseases, University of North Carolina at Chapel Hill, Chapel Hill, North Carolina, U.S.A.

**Keywords:** malaria relapse, Markov transition model, quadratic approximation, two-stage estimation

## Abstract

A standard competing risks set-up requires both time to event and cause of failure to be fully observable for all subjects. However, in application, the cause of failure may not always be observable, thus impeding the risk assessment. In some extreme cases, none of the causes of failure is observable. In the case of a recurrent episode of *Plasmodium vivax* malaria following treatment, the patient may have suffered a relapse from a previous infection or acquired a new infection from a mosquito bite. In this case, the time to relapse cannot be modeled when a competing risk, a new infection, is present. The efficacy of a treatment for preventing relapse from a previous infection may be underestimated when the true cause of infection cannot be classified. In this paper, we developed a novel method for classifying the latent cause of failure under a competing risks set-up, which uses not only time to event information but also transition likelihoods between covariates at the baseline and at the time of event occurrence. Our classifier shows superior performance under various scenarios in simulation experiments. The method was applied to *Plasmodium vivax* infection data to classify recurrent infections of malaria.

## 1 Introduction

### 1.1 *Plasmodium vivax* Malaria Infection

*Plasmodium vivax*, in short, *P. vivax*, is the most widespread human malaria (Howes et al., 2016). According to the 2019 World Malaria Report released by World Health Organization (WHO), 53% of the global *P. vivax* burden is in the South-East Asia Region, and 75% of malaria cases in the Region of the Americas are resulted from *P. vivax*. Due to the dormant liver stage of *P. vivax*, *hypnozoites* may reactivate and cause another infection weeks to months after the initial infection (Chu and White, 2016). Relapse due to inadequately treated blood stages is less common and is referred to as treatment failure or recrudescence. Therefore, when first-line anti-malarials are used, relapse is usually attributed to *hypnozoite*-induced relapse. *P. vivax* relapses are an important source of morbidity and contribute to malaria mortality (Dini et al. 2020, Robinson et al. 2015, Baird 2013). However, the fact that individuals can also become reinfected due to a new mosquito bite makes it difficult to study the anti-relapse efficacy of treatment. Previous studies have concluded that even when the level of transmission is relatively low, there is a high genetic diversity in *P. vivax* parasites within patient populations in Cambodia (Lin et al., 2013, Friedrich et al., 2016). Such genetic diversity, often resulting in multiple parasites haplotypes present in a single infection, provides an opportunity for researchers to distinguish relapse from a recurrent infection by examining the overlap of haplotypes between infections and the appearance of haplotypes associated with relapse.

**Figure 1:**
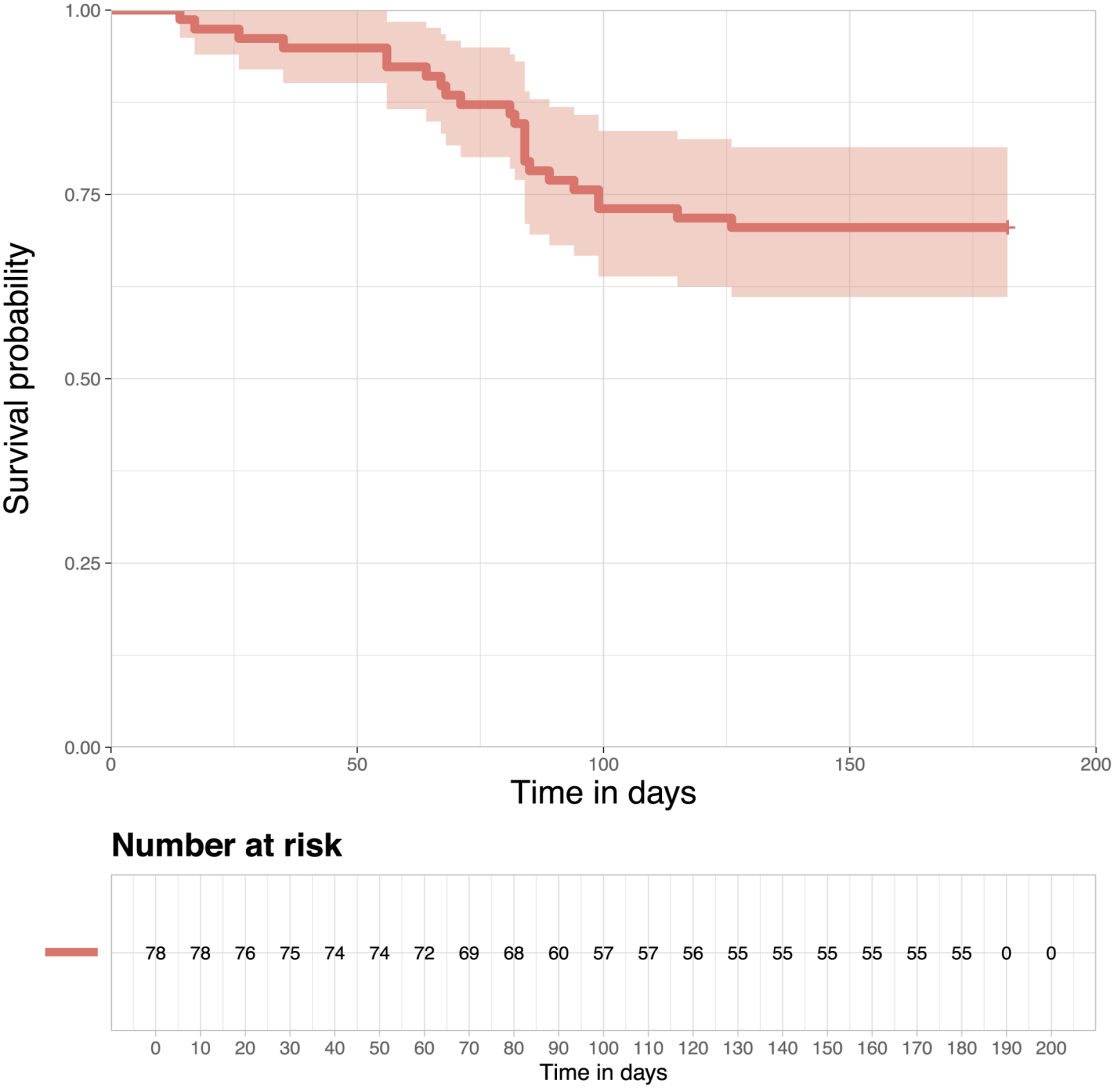
Kaplan-Meier curve for the first recurrent infection.

Lin et al. (2015) applied targeted deep sequencing to 108 isolates collected from 78 Cambodian volunteers with *P. vivax* infection (Lon et al., 2014). Subjects in the study were treated initially with dihydroartemisinin-piperaquine (DP), an effective drug to treat the blood stages of *P. vivax*, all but precluding treatment failure due to recrudescence. To detect recurrent infection, blood smears of study subjects were taken firstly at baseline, then weekly for six weeks following treatment, then monthly thereafter. At the end of the study, 23 of the 78 subjects experienced recurrent infections, with a median of 68 days in the time to recurrence. Subjects’ participation in the study ranged from 2 to 6 months, with a median of 4 months follow-up. Since treatment failure with DP is unlikely, these recurrences most likely represent relapse or reinfection. In fact, of the 23 subjects with recurrent infection, five subjects had a second recurrent infection, and one subject had a third recurrent infection. To simplify the analysis, we only consider the first recurrent infection among those 23 subjects. Figure 1 shows the Kaplan-Meier curve for the first recurrent infection along with the risk table showing the number of subjects at risk over ten-day intervals. The horizontal axis in the plot indicates days from baseline, and the vertical axis is the estimated survival probability. The solid line is the step function and shaded area is associated 95% point-wise confidence interval of the step function. The longest follow-up time is 180 days, and 70% (55 subjects) were disease-free at the end of the follow-up period. A subject-by-subject time to first infection plot is given in the Supplementary Materials.

**Figure 2:**
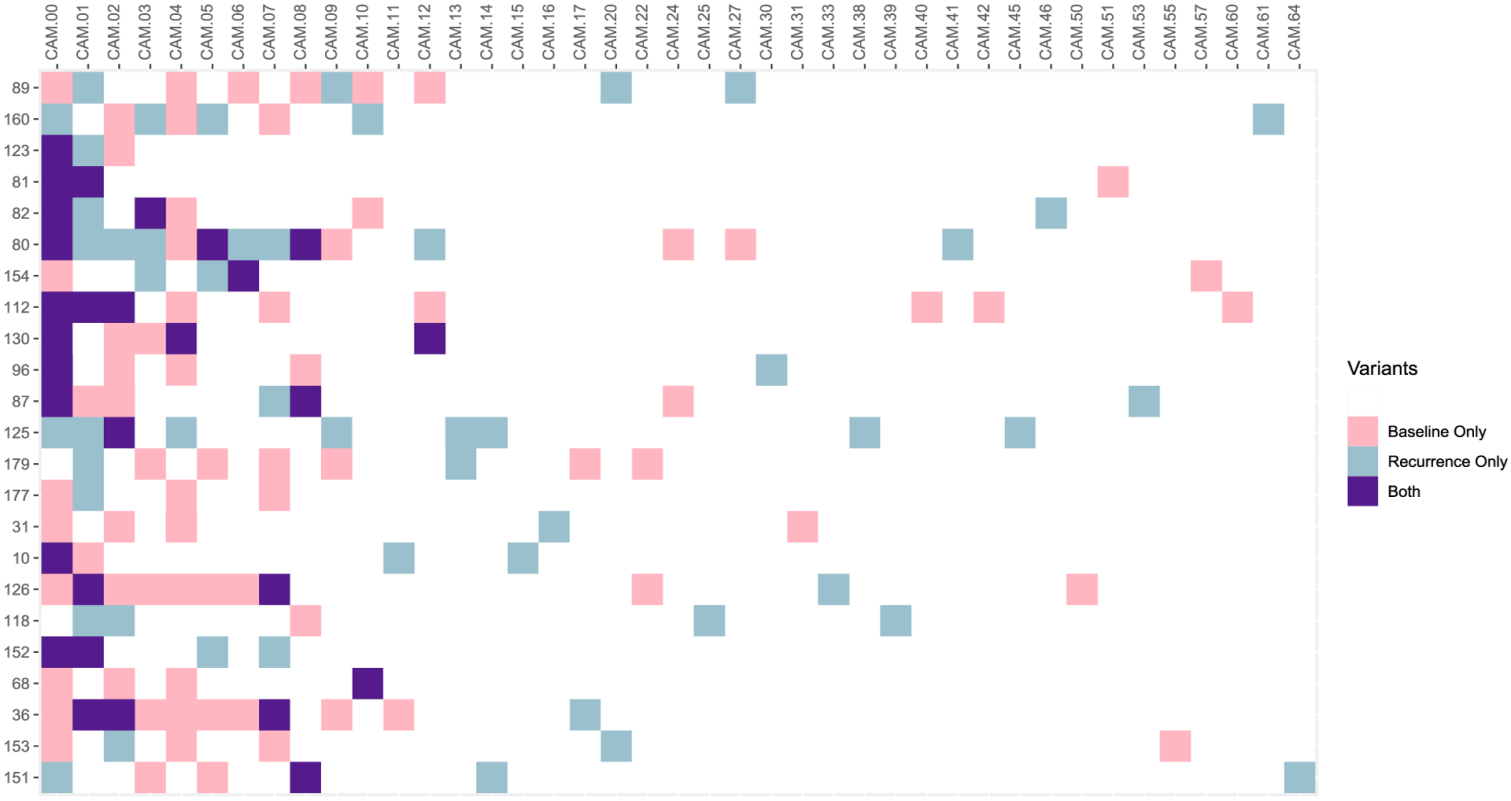
Heatmap for presence/absence of haplotypes.

*P. vivax* exhibits great genetic diversity, surpassing that seen in *P. falciparum* (Neafsey et al., 2012). Parobek et al. (2014) identified a highly variable 117-base pair (bp) segment of the *P. vivax* merozoite surface protein 1 gene (*pvmsp1*) within the 33-kDa subunit of the 42-kDa region, which exhibits great nucleotide diversity. After extracting DNA from filter paper blood spots, Lin et al. (2015) applied deep sequencing to this region and used a bioinformatics pipeline called *SeekDeep* (Hathaway et al., 2018) to determine different haplotypes of *pvmsp1* defined by at least a single nucleotide difference between haplotypes. They identified 67 unique *pvmsp1* haplotypes across 108 isolates from either initial infection or recurrent infections, with each patient isolate harboring, on average, three different haplotypes. They found nine haplotypes that are common and appeared in at least 10% of individuals. 46 rare haplotypes appeared in only one isolate, with some later attributed to sequencing error. Only 41 unique haplotypes were identified in those subjects with recurrent infection. Figure 2 shows a heatmap that indicates the presence/absence of these 41 haplotypes (genetic variants) in the initial and recurrent infections from those 23 subjects. Each column represents one unique haplotype, and each row represents one subject with an identification number. The subjects were sorted based on their time to the first recurrent infection, with the shortest time at the top and the longest time at the bottom. Pink cells indicate the presence of the haplotype in the initial infection but absence in the recurrent infection. Blue cells show the absence of the haplotype in the initial infection but presence in the recurrent infection. Purple cells show haplotypes that were present in both infections. Interestingly, only 16 subjects had overlapping haplotypes between initial and recurrent infections. Two subjects with the shortest time to recurrent infection did not have any shared haplotypes.

### 1.2 Competing Risks with Unknown Cause of Failure

It is commonly seen in biomedical research that the occurrence of an event during the follow-up period can be attributed to one of multiple causes. Data of this type is a standard competing risks set-up, where one event occurs per subject, and the failure type is one of many possible causes. Usually, both time to event and the cause of failure are observable. However, in some cases, the cause of failure may be unknown or missing. For example, in *P. vivax* malaria research, subjects who live in endemic areas suffer recurrent infections which can arise from (1) mosquito bites representing new infection, (2) relapse from latent infection in the liver, or (3) recrudescence due to treatment failure. The cause of recurrent infection is unknown or indeterminable in this case, thus impeding the efficacy assessment of anti-relapse treatment. Developing a reliable method to distinguish new infections from relapse is critical.

The problem of missing cause of failure in competing risks data has been given much attention since Dinse (1982). There are two possible approaches for estimating competing risks data with missing cause of failure when the cause is missing at random (Rubin, 1976): (1) complete-case analysis, utilizing only complete observations, e.g., Effraimidis and Dahl (2014), or, (2) construct a regression model for the missing cause using all observations, including those with missing cause of failure. In the second approach, one can use a global parametric model (Lu and Tsiatis, 2001), a semi-parametric framework (Goetghebeur and Ryan, 1995) or a nonparametric regression method (Gouskova et al., 2017) to estimate the cause-specific hazard functions. A similar problem is also considered in Sun and Gilbert (2012) and Juraska and Gilbert (2016) when considering the competing cause as a mark for the mark-specific hazard function. A doubly robust estimator is proposed in these papers when the mark variable is possibly missing. However, these approaches require at least some of the observations to have complete records. They cannot be applied to the problem in *P. vivax* malaria research, where the cause of failure is unknown for every subject.

When analyzing the causes of *P. vivax* malaria recurrence from a competing risks perspective, it is natural to assume that the time to recurrent infection is associated with baseline covariates (e.g., genetic variants or haplotypes) collected at the initial infection. We assume that each cause has a distinct cause-specific hazard function conditional on the baseline covariates, enabling us to build an initial cause classifier that can distinguish the cause based on the time to recurrence information. Subsequently, by observing changes in the values of genetic variants between initial and recurrent infections, one can build another classifier that can distinguish the cause of failure, as the changes are driven by the latent cause. Thus, one can update the initial classifier by utilizing the information contained in the transition of covariates between initial infection and recurrent infection. To study the transition mechanism, Lin et al. (2020) proposed an approach that estimates the transition likelihoods using both shared and non-shared genetic variants to improve classification accuracy when the cause of recurrent infection is unknown. Bureau et al. (2003) utilized a continuous-time hidden Markov chain to obtain the true transition probabilities between states when the disease status is possibly misclassified. However, Lin et al. (2020) did not consider the time to recurrent infection, and Bureau et al. (2003) required the disease status to be fully observed but subject to misclassification. Neither of these approaches is ideal for our malaria data, and can not be applied to the classification problem when dealing with competing risks data with missing cause of failure.

In the classification problem with unknown cause of malaria recurrence, Taylor et al. (2019) proposed a Bayesian approach that models the time to recurrent infection for prior classification probability and then computes the posterior probability based on an assumed genetic model with a strong prior assumption. Ferreira MU, de Sousa TN et al. (2020) treated relapse (combined with recrudescence) and new infection as competing risks assuming an exponential distribution with a time-constant hazard for both causes. In contrast, we analyze the time to event data under a competing risks set-up without specifying any temporal pattern of the hazard function. We generalize the idea in Lin et al. (2020) to incorporate the transition likelihoods between covariates to classify the unknown cause of infection. By considering the time to event information and transition likelihoods at the same time, we utilize more information from the data and thus lead to a more accurate classifier. Our method allows the causes of failure to be completely missing and can be applied to *P. vivax* malaria data (Lin et al., 2015). The classification procedure includes two main steps. First, we utilize the time to event and baseline covariates information to obtain an initial classifier. Then, we update the classification probability obtained in the first step using transition likelihoods between covariates to obtain the second classifier, whose performance is better than the first one. The challenges of building these classifiers are that the covariates are high-dimensional, and they can be of different kinds of variables. To resolve the first challenge, we propose a penalized maximum partial likelihood estimator and use an efficient proximal gradient descent algorithm to obtain the estimator. To resolve the second challenge, we propose a general transition likelihood that can incorporate different kinds of variables.

The rest of this paper is organized as follows. In Section 2, we describe the method of modeling competing risk data under a proportional hazards model with baseline covariates. In Section 3, we introduce general formulae for the two classifiers. An algorithm for the computation of parameters needed for constructing the classifiers is laid out in Section 4. We carry out comprehensive simulation experiments under various scenarios to evaluate the performance of the proposed classifiers in Section 5. Finally, we apply the developed method to the *P. vivax* malaria data and show the classification result in Section 6. We summarize our current approach and discuss its extensions in Section 7.

## 2 Model and Estimation

In a general setting of competing risks, let $T_{i}^{*}$ be the failure time and *ϵ*_*i*_ ∈ {1, 2} be the cause of failure for subject *i*. We consider only two causes of failure since this is the most general setting of competing risks application. If there are more than two causes, one may combine causes other than the primary interest into one category and format the model with two causes of failure. To model the time to failure when competing risks are presented, we consider a cause-specific hazard function for cause *k*, (*k* = 1, 2), defined by: $\lambda_{ik}(t)={\text{lim}}_{dt\to 0}P(t⩽T_{i}^{*}<t+dt,\epsilon_{i}=k|T_{i}^{*}⩾t)/dt$. With ***X***_*i*_ = (*X*_*i*1_, …, X_*iJ*_)′ being the *J*-dimensional vector of covariates at the baseline, we consider a proportional hazards model for the cause-specific hazard function, defined by $\lambda_{ik}(t;\beta )=\lambda_{0k}(t)\text{exp}\left({\beta_{k}}^{\prime }X_{i}\right)$, where *λ*_0*k*_(*t*) is the baseline hazard function for cause *k*, ***β***_*k*_ = (*β*_*i*1_, …, *β*_*kJ*_)′ is the vector of regression coefficients, and $\beta ={\left(\beta_{1}^{\prime },\beta_{2}^{\prime }\right)}^{\prime }$ (Kalbfleisch and Prentice, 2002, Section 8.2).

When the causes of failure are fully observed and time to failure is right-censored, one observes $T_{i}=\text{min}\left(T_{i}^{*},C_{i}\right),\delta_{i}=I\left(T_{i}⩽C_{i}\right)$, and failure type *ϵ*_*i*_ when *δ*_*i*_ = 1, where *I*(·) is the indicator function. Assume {*T*_*i*_, *δ*_*i*_, *δ*_*i*_*ϵ*_*i*_, ***X***_*i*_} are i.i.d. for *i* = 1, …, *n*. Under the fully observed data, we estimate ***β*** using the partial likelihood function

(1) $$\prod_{i=1}^{n}\prod_{k=1}^{2}{\left\{\frac{\text{exp}\left({\beta_{k}}^{\prime }X_{i}\right)}{\sum_{l\in R_{i}}\text{exp}\left({\beta_{k}}^{\prime }X_{l}\right)}\right\}}^{\delta_{ik}},$$

where *δ*_*ik*_ = *δ*_*i*_*I*(*ϵ*_*i*_ = *k*) indicates whether the failure of cause *k* occurs, and *R*_*i*_ ≡ {*l*: *T*_*l*_ ⩾ *T*_*i*_} is a set of subjects who are at risk at *T*_*i*_. However, in our case, *neither* cause was observed. Thus, the partial likelihood function above is not feasible since *δ*_*ik*_ is not observable. When neither cause is observed, the available data is {*T*_*i*_, *δ*_*i*_, ***X***_*i*_} for *i* = 1, …, *n*, which is identical to the conventional right-censoring time to event data. The partial likelihood function for ***β*** is

(2) $$\prod_{i=1}^{n}{\left\{\frac{\lambda_{i}\left(T_{i}\right)}{\sum_{\ell \in R_{i}}\lambda_{\ell }\left(T_{i}\right)}\right\}}^{\delta_{i}},$$

where *λ*_*i*_(*t*) is the overall hazard function. Assuming only one event can occur at time *t* + *dt*, one writes the overall hazard function as $\lambda_{i}(t)=\sum_{k=1}^{2}\lambda_{ik}(t)$ since $P\left(t⩽T_{i}^{*}<t+dt|T_{i}^{*}⩾t\right)=\sum_{k=1}^{2}P\left(t⩽T_{i}^{*}<t+dt,\epsilon_{i}=k|T_{i}^{*}⩾t\right)$. Hence, (2) becomes

$$\prod_{i=1}^{n}{\left\{\frac{\sum_{k=1}^{2}\lambda_{0k}\left(T_{i}\right)\text{exp}\left({\beta_{k}}^{\prime }X_{i}\right)}{\sum_{\ell \in R_{i}}\sum_{k=1}^{2}\lambda_{0k}\left(T_{i}\right)\text{exp}\left({\beta_{k}}^{\prime }X_{\ell }\right)}\right\}}^{\delta_{i}},$$

where the baseline hazard function *λ*_0*k*_(*t*) cannot be completely unspecified for *k* = 1, 2, unlike the partial likelihood function in (1).

The primary interest of the competing risks model in our application is written as

(3) $$\lambda_{i1}(t)=\lambda_{0}(t)\text{exp}(\alpha ),$$

(4) $$\lambda_{i2}(t)=\lambda_{0}(t)\text{exp}\left(\beta^{\prime }X_{i}\right).$$

This model fits naturally with the *P. vivax* malaria data we intend to analyze. Reinfection is considered as the first cause of failure (*ϵ*_*i*_ = 1) that randomly occurs from the environment following a time-to-event distribution with no association with the baseline covariates ***X***_*i*_. We assume its hazard *λ*_*i*1_(*t*) can be written as the baseline hazard *λ*_0_(*t*) attenuated by a constant factor exp(*α*) as shown in model (3). The hazard function *λ*_*i*1_(*t*) is considered as the background hazard. For the *P. vivax* malaria study, *λ*_*i*1_(*t*) represents a random mosquito bite from the living or working environment. Relapse is considered the second cause of failure (*ϵ*_*i*_ = 2) that is associated with the baseline covariates ***X***_*i*_ in model (4), which follows a proportional hazards model. These two causes of failure compete to occur, and only one of the causes, either relapse or reinfection, would occur if the event time is not censored. Under models (3) and (4), both hazard functions share the same baseline hazard *λ*_0_(*t*). The ratio of *λ*_*i*1_(*t*) and *λ*_*i*2_(*t*) only depends on baseline covariates ***X***_*i*_, and can be considered as a semiparametric two-sample density ratio model promoted by Qin (1998). The baseline hazard *λ*_0_(*t*) here needs no specification, and can be any function of time. It can also be a function of covariates, under the condition that covariates included in *λ*_0_(*t*) are independent of those in ***X***_*i*_.

Without any specification of *λ*_0_(*t*), one can use the partial likelihood function

(5) $$PL(\theta )=\prod_{i=1}^{n}{\left[\frac{\text{exp}(\alpha )+\text{exp}\left(\beta^{\prime }X_{i}\right)}{\sum_{\ell \in R_{i}}\left\{\text{exp}(\alpha )+\text{exp}\left(\beta^{\prime }X_{\ell }\right)\right\}}\right]}^{\delta_{i}},$$

to estimate ***θ*** = (*α*, ***β***′)′ where *α* and ***β*** are unknown parameters of interest. However, the dimensionality of ***θ*** is a concern in our case since genetic sequencing produces a large number of haplotypes that are considered as covariates in our model. In Section 4, we introduce a penalized maximum partial likelihood method to estimate the high-dimensional ***θ***.

In addition, we discuss an approach to verify the specification of models (3) and (4) for the *P. vivax* malaria data. The model diagnosis can be explored by martingale residuals defined by ${\hat{M}}_{i}=\delta_{i}-{\hat{\Lambda }}_{i}\left(T_{i}\right)$ for subjects *i* = 1, …, *n*, where ${\hat{\Lambda }}_{i}(t)$ is the estimated cumulative hazard function for Λ_*i*_(*t*) = Λ_0_(*t*){exp(*α*) + exp(***β***′***X****_i_*)}. The estimation involves not only parameter estimates for ***θ*** = (*α*, ***β***′)′, but also baseline hazard estimate for $\Lambda_{0}(t)=\int_{0}^{t}\lambda_{0}(s)ds$. One can use a Breslow-type estimator ${\hat{\Lambda }}_{0}(t)=\sum_{i=1}^{n}I\left(T_{i}⩽t\right)\delta_{i}/\sum_{j\in R_{i}}\left\{\text{exp}(\hat{\alpha })+\text{exp}\left({\hat{\beta }}^{\prime }X_{j}\right)\right\}$ for Λ_0_(*t*) and calculate a test statistic $T(x)=\sum_{i=1}^{n}I\left({\hat{\beta }}^{\prime }X_{i}⩽x\right){\hat{M}}_{i}$ for a lack-of-fit test over the follow-up time. One can construct a confidence band for *T*(*x*) via Monte-Carlo simulation, as proposed in Lin et al. (1993). Model diagnosis results for the *P. vivax* malaria data are given in Section 6.

## 3 Classification

We propose two classifiers to classify the event to one of the two causes. The first classifier uses the baseline information and partial likelihood function (5) to obtain the initial estimate of the probability that the event is of cause *k*. The second classifier updates the first classifier using transition likelihoods under different causes. We expect that the second classifier will perform better when the transition of covariates is informative since more information is involved. If the transition of covariates is not informative of the cause of failure, the second classifier improves little from the first classifier.

### 3.1 Based on Baseline Information

Let $N_{i}^{*}(t)$ be the number of events up to time *t*, and $dN_{i}^{*}(t)=N_{i}^{*}(t+dt)-N_{i}^{*}(t)$ be the event indicator in the next instantaneous time *dt* after *t*. The observed counting process is $N_{i}(t)=Y_{i}(t)N_{i}^{*}(t)$, where *Y*_i_(*t*) = *I* (*T*_*i*_ ⩾ *t*) indicates whether subject *i* is at risk at time *t*. Let $\xi_{ik}^{\left(0\right)}(t)=P\left(\epsilon_{i}=k|d\;N_{i}(t)=1,X_{i}=x_{i}\right)$ be the probability of cause *k*, given that an event occurs in [*t*, *t* + *dt*) and the realization of baseline covariate is ***X***_*i*_ = ***x***_*i*_. We have: $\xi_{ik}^{\left(0\right)}(t)=P(\epsilon_{i}=k|d\;N_{i}(t)=1,X_{i}=x_{i})=\lambda_{ik}(t;\theta )/\lambda_{i}(t;\theta )$. If an event occurs at *T*_*i*_ = *t*_*i*_ for subject *i*, $\xi_{ik}^{\left(0\right)}\left(t_{i}\right)$ can be estimated by

(6) $${\hat{\xi }}_{i1}^{\left(0\right)}\left(t_{i}\right)=\frac{\lambda_{i1}\left(T_{i};\hat{\theta }\right)}{\lambda_{i}\left(T_{i};\hat{\theta }\right)}=\frac{\lambda_{0}\left(T_{i}\right)\text{exp}(\hat{\alpha })}{\lambda_{0}\left(T_{i}\right)\left\{\text{exp}(\hat{\alpha })+\text{exp}\left({\hat{\beta }}^{\prime }x_{i}\right)\right\}}=\frac{\text{exp}(\hat{\alpha })}{\text{exp}(\hat{\alpha })+\text{exp}\left({\hat{\beta }}^{\prime }x_{i}\right)},$$

(7) $${\hat{\xi }}_{i2}^{\left(0\right)}\left(t_{i}\right)=\frac{\lambda_{i2}\left(T_{i};\hat{\theta }\right)}{\lambda_{i}\left(T_{i};\hat{\theta }\right)}=\frac{\lambda_{0}\left(T_{i}\right)\text{exp}\left({\hat{\beta }}^{\prime }x_{i}\right)}{\lambda_{0}\left(T_{i}\right)\left\{\text{exp}(\hat{\alpha })+\text{exp}\left({\hat{\beta }}^{\prime }x_{i}\right)\right\}}=\frac{\text{exp}\left({\hat{\beta }}^{\prime }x_{i}\right)}{\text{exp}(\hat{\alpha })+\text{exp}\left({\hat{\beta }}^{\prime }x_{i}\right)},$$

where $\hat{\theta }$ is the maximum partial likelihood estimator of ***θ*** in (5). Since formulae (6) and (7) are independent of *t*_*i*_, we write ${\hat{\xi }}_{i1}^{\left(0\right)}$ and ${\hat{\xi }}_{i2}^{\left(0\right)}$ in short for ${\hat{\xi }}_{i1}^{\left(0\right)}\left(t_{i}\right)$ and ${\hat{\xi }}_{i2}^{\left(0\right)}\left(t_{i}\right)$, respectively.

We classify an event to be of cause 2 if ${\hat{\xi }}_{i2}^{\left(0\right)}>{\hat{\xi }}_{i1}^{\left(0\right)}$ and to be of cause 1 otherwise.

### 3.2 Based on Both Baseline and Event Information

When an event occurs for subject *i*, we assume that ***Z***_*i*_ = (*Z*_*i*1_, …, *Z*_*iJ*_)′ is collected at the event time, which is the same set of covariates as baseline covariates ***X***_*i*_. We propose to utilize the transitions from ***X***_*i*_ to ***Z***_*i*_ to aid the cause classification. Let $\xi_{ik}^{\left(1\right)}(t)=P(\epsilon_{i}=k|dN_{i}(t)=1,X_{i}=x_{i},Z_{i}=z_{i})$ be the probability of cause *k* given realizations of both ***X***_*i*_ = ***x***_*i*_ and ***Z***_*i*_ = ***z***_*i*_. One can show that

$$\xi_{ik}^{\left(1\right)}(t)=\frac{f\left(z_{i}|\epsilon_{i}=k,dN_{i}(t)=1,X_{i}=x_{i}\right)P\left(\epsilon_{i}=k|dN_{i}(t)=1,X_{i}=x_{i}\right)}{\sum_{k=1}^{2}f\left(z_{i}|\epsilon_{i}=k,dN_{i}(t)=1,X_{i}=x_{i}\right)P\left(\epsilon_{i}=k|dN_{i}(t)=1,X_{i}=x_{i}\right)}\;=\frac{\phi_{i}(k)\xi_{ik}^{\left(0\right)}(t)}{\sum_{k=1}^{2}\phi_{i}(k)\xi_{ik}^{\left(0\right)}(t)},$$

where *ϕ*_*i*_(*k*) = *f*(*z*_*i*_|*ϵ*_*i*_ = *k*, *dN*_*i*_(*t*) = 1, ***X***_*i*_ = ***x***_*i*_) is the conditional density function of ***Z***_*i*_ given ***X***_*i*_ under cause *k*. We call *ϕ*_*i*_(*k*) the conditional *transition likelihood* of cause *k*. One can treat the classification probability $\xi_{ik}^{\left(1\right)}(t)$ as an updated version of $\xi_{ik}^{\left(0\right)}(t)$ by the ratio of transition likelihoods between possible causes since $\frac{\xi_{ik}^{\left(1\right)}(t)}{\xi_{i\ell }^{\left(1\right)}(t)}=\frac{\phi_{i}(k)}{\phi_{i}(\ell )}\frac{\xi_{ik}^{\left(0\right)}(t)}{\xi_{i\ell }^{\left(0\right)}(t)}$ for ℓ = 1, 2 and *ℓ* ≠ *k*. Note that if the transition likelihoods are informative, *ϕ*_*i*_(1) and *ϕ*_*i*_(2) will be very different from each other and thus lead to a more accurate classification of $\xi_{ik}^{\left(1\right)}(t)$.

We assume that the transition likelihood *ϕ*_*i*_(*k*) follows a parametric model *ϕ*_*i*_(*k*, ***γ***_*k*_), where ***γ***_*k*_ is the vector of parameters to be estimated. More details of this parametric model *ϕ*_*i*_(*k*) follow in Section 3.3. The distribution of ***Z***_*i*_ is a mixture of transition likelihoods from two latent causes:

$$f\left(z_{i}|dN_{i}(t)=1,X_{i}=x_{i}\right)=\sum_{k=1}^{2}f\left(z_{i},\epsilon_{i}=k|dN_{i}(t)=1,X_{i}=x_{i}\right)\;=\sum_{k=1}^{2}f\left(z_{i}|\epsilon_{i}=k,dN_{i}(t)=1,X_{i}=x_{i}\right)P\left(\epsilon_{i}=k|dN_{i}(t)=1,X_{i}=x_{i}\right)\;=\sum_{k=1}^{2}\phi_{i}\left(k,\gamma_{k}\right)\xi_{ik}^{\left(0\right)}(t).$$

With $\xi_{ik}^{\left(0\right)}(t)$ being estimated by ${\hat{\xi }}_{ik}^{\left(0\right)}$, and let $m=\sum_{i=1}^{n}\delta_{i}$ be the number of subjects having recurrent infections. We estimate ***γ***_*k*_ by maximizing a pseudo log-likelihood function:

(8) $$\ell \left(\gamma_{1},\gamma_{2}\right)=\sum_{i=1}^{m}\text{log}\left\{\sum_{k=1}^{2}\phi_{i}\left(k,\gamma_{k}\right){\hat{\xi }}_{ik}^{\left(0\right)}\right\}.$$

Let ${\left({\hat{\gamma }}_{1}^{\prime },{\hat{\gamma }}_{2}^{\prime }\right)}^{\prime }={\text{argmax}}_{\gamma_{1},\gamma_{2}}\ell \left(\gamma_{1},\gamma_{2}\right)$ and write ${\hat{\xi }}_{ik}^{\left(1\right)}$ in short for ${\hat{\xi }}_{ik}^{\left(1\right)}\left(t_{i}\right)$. We estimate $\xi_{ik}^{\left(1\right)}$ by

(9) $${\hat{\xi }}_{ik}^{\left(1\right)}=\frac{\phi_{i}\left(k,{\hat{\gamma }}_{k}\right){\hat{\xi }}_{ik}^{\left(0\right)}}{\sum_{k=1}^{2}\phi_{i}\left(k,{\hat{\gamma }}_{k}\right){\hat{\xi }}_{ik}^{\left(0\right)}}.$$

We classify the event to be of cause 2 if and only if ${\hat{\xi }}_{i2}^{\left(1\right)}>{\hat{\xi }}_{i1}^{\left(1\right)}$.

### 3.3 Transition Likelihood

The transition likelihood plays a critical role in classification. In this section, we discuss a generalized linear model to model the transition likelihood function *ϕ*_*i*_(*k*, ***γ***_*k*_). Suppose the density of *Z*_*ij*_ conditioning on *X*_*ij*_ and *ϵ*_*i*_ = *k* has the form of

$$f\left(z;\vartheta_{ijk},\psi_{jk}\right)=\text{exp}\left\{\left(z\vartheta_{ijk}-b\left(\vartheta_{ijk}\right)\right)/a\left(\psi_{jk}\right)+c\left(z,\psi_{jk}\right)\right\},$$

where *a*(·), *b*(·) and *c*(·) are known functions, *ϑ*_*ijk*_ is the natural parameter, and *ψ*_*jk*_ is the dispersion parameter (McCullagh and Nelder, 1989). Let *g*(*μ*_*ijk*_) = *ϑ*_*ijk*_ be the cause-specific canonical link function, where *μ*_*ijk*_ = E(*Z*_*ij*_|*ϵ*_*i*_ = *k*, *dN*_*i*_(*t*) = 1, *X*_*ij*_ = *x*_*ij*_). We define *ϕ*_*i*_(*k*, ***γ***_*k*_) as:

$$\phi_{i}\left(k,\gamma_{k}\right)=f\left(z_{i}|\epsilon_{i}=k,dN_{i}(t)=1,X_{i}=x_{i}\right)\;=\prod_{j=1}^{J}\text{exp}\left[\left\{z_{ij}g\left(\mu_{ijk}\right)-b\left(g\left(\mu_{ijk}\right)\right)\right\}/a\left(\psi_{jk}\right)+c\left(z_{ij},\psi_{jk}\right)\right],$$

where *g*(*μ*_*ijk*_) = *q*_*jk*0_ + *x*_*ij*_*q*_*jk*1_, *q*_*jk*0_ is the intercept term and *q*_*jk*1_ is the coefficient of *x*_*ij*_.

To improve the classification performance, we want the transition likelihoods to be as informative as possible. When some external variables contain information about the transition, we also would like to incorporate them into the transition likelihoods. Let ***W***_*ij*_ = (*W*_*ij*1_, *W*_*ij*2_, …, *W*_*ijL*_)′ be the *L*-dimensional vector of these external variables and $W_{i}={\left(W_{i1}^{\prime },W_{i2}^{\prime },\ldots ,W_{iJ}^{\prime }\right)}^{\prime }$. Then, we have

$$\phi_{i}\left(k,\gamma_{k}\right)=f\left(z_{i}|\epsilon_{i}=k,dN_{i}(t)=1,X_{i}=x_{i},W_{i}=w_{i}\right)\;=\prod_{j=1}^{J}\text{exp}\left[\left\{z_{ij}g\left(\mu_{ijk}\right)-b\left(g\left(\mu_{ijk}\right)\right)\right\}/a\left(\psi_{jk}\right)+c\left(z_{ij},\psi_{jk}\right)\right],$$

where $g\left(\mu_{ijk}\right)=q_{jk0}+x_{ij}q_{jk1}+w_{ij}^{\prime }q_{jk}^{*}$, ***w***_*ij*_ is a realization of ***W***_*ij*_ with the corresponding coefficients $q_{jk}^{*}={\left(q_{jk1}^{*},q_{jk2}^{*},\ldots ,q_{jkL}^{*}\right)}^{\prime }$. Let ***q***_*k*0_ = (*q*_1*k*0_, …, *q*_*Jk*0_)′, ***q***_*k*1_ = (*q*_1*k*1_, …, *q*_*Jk*1_)′, $q_{k}^{*}={\left(q_{1k}^{*}{}^{\prime },\ldots ,q_{Jk}^{*}{}^{\prime }\right)}^{\prime }$ and ***ψ***_*k*_ = (*ψ*_1*k*_, …, *ψ*_*Jk*_)′. Then, we let $\gamma_{k}={\left(q_{k0}^{\prime },q_{k1}^{\prime },q_{k}^{{*}^{\prime }},\psi_{k}^{\prime }\right)}^{\prime }$ represent all the parameters in *ϕ*_*i*_(*k*, ***γ***_*k*_).

Our proposed transition likelihood model manifests differently according to the type of covariates. We give three examples showing how to construct *ϕ*_*i*_(*k*, ***γ***_*k*_) when the covariates are binary, normal, or Poisson.

**Example 1** (Binary Covariates). *When X*_*ij*_ *and Z*_*ij*_ *are binary covariates, we have*

$$g\left(\mu_{ijk}\right)=\text{log}\left(\frac{\mu_{ijk}}{1-\mu_{ijk}}\right)=q_{jk0}+x_{ij}q_{jk1}+w_{ij}^{\prime }q_{jk}^{*},$$

*where the link function g is a logit function. The transitional likelihood for cause k becomes*

(10) $$\phi_{i}\left(k,\gamma_{k}\right)=\prod_{j=1}^{J}\mu_{ijk}^{z_{ij}}{\left(1-\mu_{ijk}\right)}^{1-z_{ij}},$$

*where μ*_*ijk*_ = exp(*ϑ*_*ijk*_)/{1 + exp(*ϑ*_*ijk*_)}, $\vartheta_{ijk}=q_{jk0}+x_{ij}q_{jk1}+w_{ij}^{\prime }q_{jk}^{*},\gamma_{k}={\left(q_{k0}^{\prime },q_{k1}^{\prime },q_{k}^{{*}^{\prime }}\right)}^{\prime }$ *and* ***ψ***_*k*_ = (1, …, 1)′.

**Example 2** (Normal Covariates). *When X*_*ij*_ *and Z*_*ij*_ *are normally distributed covariates, we have*

$$g\left(\mu_{ijk}\right)=\mu_{ijk}=q_{jk0}+x_{ij}q_{jk1}+w_{ij}^{\prime }q_{jk}^{*},$$

$$\psi_{jk}=Var\left(Z_{ij}|\epsilon_{i}=k,dN_{i}(t)=1,X_{ij}=x_{ij},W_{ij}=w_{ij}\right),$$

*where the link function g is an identity function. The transitional likelihood for cause k becomes*

(11) $$\phi_{i}\left(k,\gamma_{k}\right)=\prod_{j=1}^{J}\frac{1}{\sqrt{2\pi \psi_{jk}}}\text{exp}\left\{-\frac{{\left(z_{ij}-\mu_{ijk}\right)}^{2}}{2\psi_{jk}}\right\},$$

where $\gamma_{k}={\left(q_{k0}^{\prime },q_{k1}^{\prime },q_{k}^{{*}^{\prime }},\psi_{k}^{\prime }\right)}^{\prime }$ and ***ψ***_*k*_ = (*ψ*_1*k*_, …, *ψ*_*Jk*_)′.

**Example 3** (Poisson Covariates). *When X*_*ij*_ *and Z*_*ij*_ *are Poisson covariates, we have*

$$g\left(\mu_{ijk}\right)=\text{log}\left(\mu_{ijk}\right)=q_{jk0}+x_{ij}q_{jk1}+w_{ij}^{\prime }q_{jk}^{*},$$

*where the link function g is a log function. The transitional likelihood for cause k becomes*

(12) $$\phi_{i}\left(k,\gamma_{k}\right)=\prod_{j=1}^{J}\frac{\mu_{ijk}^{z_{ij}}\text{exp}\left(-\mu_{ijk}\right)}{z_{ij}!},$$

*where μ*_*ijk*_ = exp(*ϑ*_*ijk*_), $\vartheta_{ijk}=q_{jk0}+x_{ij}q_{jk1}+w_{ij}^{\prime }q_{jk}^{*},\gamma_{k}={\left(q_{k0}^{\prime },q_{k1}^{\prime },q_{k}^{{*}^{\prime }}\right)}^{\prime }$ *and* ***ψ***_*k*_ = (1, …, 1)′.

## 4 Computation

### 4.1 Estimation of Parameters

Define the negative partial log-likelihood function as

(13) $$\ell (\theta )=-\sum_{i=1}^{n}\delta_{i}\left[\text{log}\left\{\text{exp}(\alpha )+\text{exp}\left(\beta^{\prime }X_{i}\right)\right\}-\text{log}\left\{\sum_{l\in R_{i}}\left\{\text{exp}(\alpha )+\text{exp}\left(\beta^{\prime }X_{l}\right)\right\}\right\}\right].$$

To estimate ***θ*** in (5), we propose to solve a penalized partial likelihood problem

(14) $$\hat{\theta }=\underset{\theta }{\text{argmin}}\;\{\ell (\theta )+vp(\beta )\},$$

where *ν* is a positive tuning parameter and *p*(***β***) is a penalty function. When sample size *n* is larger than the number of covariates *J*, (14) is a low-dimensional problem, in which case we set *ν* = 0. When *n* is smaller than *J*, (14) is a high-dimensional problem, in which case we choose the optimal *ν* by minimizing the Bayesian Information Criterion (BIC, Schwarz, 1978), which is given by $\text{BIC}=2\ell (\hat{\theta })+c\cdot \text{log}(n)$, where *c* is the number of covariates selected in the model. Popular choices of *p*(***β***) include the *L*_1_-penalty (Tibshirani, 1996), the elastic net penalty (Zou and Hastie, 2005), or some folded concave penalty (Fan and Lv, 2011). In this paper, we choose the *L*_1_-penalty.

To solve (14), we use a proximal gradient algorithm (Parikh and Boyd, 2014). First, we find a quadratic approximation to *ℓ*(***θ***) centered at ***θ***^(*h*)^, the estimate of ***θ*** at the *h*th iteration of the algorithm, that majorizes *ℓ*(***θ***). That is

(15) $$\ell (\theta )⩽\ell \left(\theta^{\left(h\right)}\right)+{\left(\theta -\theta^{\left(h\right)}\right)}^{\prime }\nabla \ell \left(\theta^{\left(h\right)}\right)+\frac{1}{2d}{\left\|\theta -\theta^{\left(h\right)}\right\|}_{2}^{2},$$

where *d* is a scalar that plays the role as a step size, $\theta^{\left(h\right)}={\left(\alpha^{\left(h\right)},\beta^{{\left(h\right)}^{\prime }}\right)}^{\prime }$ and the gradient vector ∇*ℓ*(***θ***^(*h*)^) is given by ∇*ℓ*(***θ***^(*h*)^) = (∇_*α*_*ℓ*(***θ***^(*h*)^), ∇_***β***_*ℓ*(***θ***^(*h*)^)′)′, where

(16) $$\nabla_{\alpha }\ell (\theta )=-\sum_{i=1}^{n}\delta_{i}\left[\frac{\text{exp}(\alpha )}{\text{exp}(\alpha )+\text{exp}\left(\beta^{\prime }X_{i}\right)}-\frac{\sum_{l\in R_{i}}\text{exp}(\alpha )}{\sum_{l\in R_{i}}\left\{\text{exp}(\alpha )+\text{exp}\left(\beta^{\prime }X_{l}\right)\right\}}\right],$$

(17) $$\nabla_{\beta }\ell (\theta )=-\sum_{i=1}^{n}\delta_{i}\left[\frac{X_{i}\text{exp}\left(\beta^{\prime }X_{i}\right)}{\text{exp}(\alpha )+\text{exp}\left(\beta^{\prime }X_{i}\right)}-\frac{\sum_{l\in R_{i}}\left\{X_{l}\text{exp}\left(\beta^{\prime }X_{l}\right)\right\}}{\sum_{l\in R_{i}}\left\{\text{exp}(\alpha )+\text{exp}\left(\beta^{\prime }X_{l}\right)\right\}}\right].$$

Denote the right-hand side of (15) by *Q*_*d*_(***θ***; ***θ***^(*h*)^) and let *g*(***β***) = *νp*(***β***). Then we minimize *Q*_*d*_(***θ***, ***θ***^(*h*)^) + *g*(***β***), which gives the proximal problem

(18) $$\alpha^{\left(h+1\right)}=\underset{\alpha }{\text{argmin}}\frac{1}{2}{\left\|\alpha -\left[\alpha^{\left(h\right)}-d\nabla_{\alpha }\ell \left(\theta^{\left(h\right)}\right)\right]\right\|}_{2}^{2},$$

(19) $$\beta^{\left(h+1\right)}=\underset{\beta }{\text{argmin}}\frac{1}{2}{\left\|\beta -\left[\beta^{\left(h\right)}-d\nabla_{\beta }\ell \left(\theta^{\left(h\right)}\right)\right]\right\|}_{2}^{2}+dg(\beta ).$$

The solution of (18) is given by *α*^(*h*+1)^ = *α*^(*h*)^ – *d*∇_*α*_*ℓ*(***θ***^(*h*)^). The solution of (19) is given by a proximal operator ***β***^(*h*+1)^ = prox_*dg*_(***β***^(*h*)^ – *d*∇_***β***_*ℓ*(***θ***^(*h*)^). Depending on the choice of penalty function, such an operator has a closed-form expression. For example, if we use an *L*_1_-penalty: *p*(***β***) = ∥***β***∥_1_, then prox_*dg*_(***β***^(*h*)^ − *d*∇_***β***_*ℓ*(***θ***^(*h*)^)) = *s*(***β***^(*h*)^ − *d*∇_***β***_*ℓ*(***θ***^(*h*)^)), *νd*), where *s*(***x***, *π*) is the elementwise soft-thresholding operator, whose *j*th element is defined as *s*(***x***, *π*)_*j*_ = sgn(*x*_*j*_)(|*x*_*j*_| − *π*)_+_. As for the step size, we follow Parikh & Boyd (2014, Section 4.2) and perform a backtracking line search; namely, we iteratively decrease step size until the majorization holds, i.e., the inequality (15) holds. This strategy is commonly used in the proximal gradient method.

We stop iterating the algorithm when the change in the objective function between two consecutive iterations is less than *ζ*% of the objective function’s value at the former iteration, where *ζ* ∈ (0, 100) is a user-defined stopping threshold, which we choose to be 10. A detailed algorithm is summarized as follows:


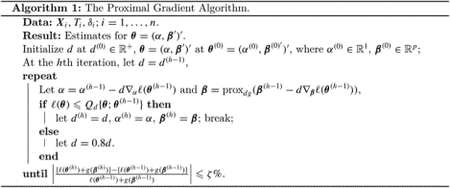


### 4.2 Classification Algorithm

We give a complete algorithm for classifying the causes of an event by using the time to event information *T*_*i*_ and *δ*_*i*_, baseline covariates ***X***_*i*_, covariates collected when the event occurs ***Z***_*i*_, and external informative covariates ***W***_*i*_ in this section.

Firstly, Given ***X***_*i*_, *T*_*i*_, and *δ*_*i*_, estimate ***θ*** using partial likelihood (5) and Algorithm 1. Secondly, estimate $\xi_{ik}^{\left(0\right)}$ by (6) and (7). Thirdly, based on the type of covariates ***X***_*i*_ and ***Z***_*i*_, estimate the *transition likelihood ϕ*_*i*_(*k*, ***γ***_*k*_) of cause *k* by maximizing the pseudo-likelihood function in (8). Next, estimate $\xi_{ik}^{\left(1\right)}$ as in (9). Finally, if ${\hat{\xi }}_{i2}^{\left(1\right)}>{\hat{\xi }}_{i1}^{\left(1\right)}$, then the event is classified as cause 2; otherwise, the event is classified as cause 1.

## 5 Simulation Experiments

To study the improvement in classification using transition likelihoods compared with using baseline information alone, we carry out comprehensive simulation experiments to evaluate the performance of two classifiers based on ${\hat{\xi }}_{i}^{\left(0\right)}$ and ${\hat{\xi }}_{i}^{\left(1\right)}$ respectively. We evaluate the performance of the proposed classifiers by comparing their sensitivity, specificity, and overall accuracy in classifying the causes of events. We mimic the data observed in the *P. vivax* malaria infection study (Lin et al., 2015) and assume that the cause could be either reinfection (*ϵ*_*i*_ = 1) or relapse (*ϵ*_*i*_ = 2). Sensitivity is defined as the number of subjects correctly classified as relapse divided by the number of relapse subjects; specificity is defined as the number of subjects correctly classified as reinfection divided by the number of reinfection subjects and overall accuracy is defined as the number of correctly classified subjects divided by the number of subjects.

Following the proposed model in Section 2, we assume that the baseline hazard is a homogeneous Poisson process with hazard function *λ*_0_(*t*), which is a constant for *t* > 0 and the same for all subjects. Using the partial likelihood function (5), we do not need to specify *λ*_0_(*t*) and expect the classification performance to be similar under different baseline hazard functions. We carry out simulations with three different baseline hazard functions *λ*_0_(*t*) = exp(*τ*), where *τ* = −0.5, 0, 0.5.

The reinfection process was assumed to be the same for all subjects with hazard function *λ*_*i*1_(*t*) = *λ*_0_(*t*) exp(*α*). The relapse process was assumed to have a proportional hazard function *λ*_*i*2_(*t*) = *λ*_0_(*t*) exp(***β***′***X***_*i*_) for subject *i*. The first classifier classifies a recurrent infection as a relapse if ${\hat{\xi }}_{i2}^{\left(0\right)}>0.5$, and the second classifier classifies a recurrent infection as a relapse if ${\hat{\xi }}_{i2}^{\left(1\right)}>0.5$.

We consider two situations where ***X***_*i*_ and ***Z***_*i*_ are binary and normally distributed variables. We allow dimensions of ***X***_*i*_ and ***Z***_*i*_ to be either low or high. Under the low-dimensional settings, we set two combinations for *n* and *J*, with (*n*, *J*) = (400, 10) and (*n*, *J*) = (800, 20). For the high-dimensional settings, we focus on the classification performance of the classifiers, as well as the variable selection performance. We consider (*n*, *J*) = (100, 200) and (*n*, *J*) = (200, 400), where the former is closer to the real *P. vivax* malaria infection study. When evaluating the variable selection performance, we focus on the sensitivity, specificity, and overall accuracy of selecting covariates with non-zero regression coefficients.

Remark that the improvement of the second classifier is mainly attributed to including the transition likelihoods from the baseline covariates ***X***_*i*_ to the covariates at recurrence infection ***Z***_*i*_. If ***Z***_*i*_ associates with ***X***_*i*_, the transition likelihood is informative, and the second classifier would have a better classification performance. However, when ***Z***_*i*_ is not associated with ***X***_*i*_, then little information would be contained in the transition likelihood. Thus, the second classifier would have a similar performance to the first classifier. We consider two scenarios where the association between ***Z***_*i*_ and ***X***_*i*_ is either strong or weak. For simplicity, we assume that for each pair of *X*_*ij*_ and *Z*_*ij*_, only one external covariate *W*_*ij*_ is associated with the transition.

### 5.1 Binary Covariates

For the low-dimensional setting, we set *α* to be 0, the first 3 components of ***β*** to be log(1.5), and the rest of the components to be 0. We generated ***X***_*i*_ from the Bernoulli distribution with probability *P*(*X*_*ij*_ = 1) = 0.5 exp{−0.1(*j* − 1)} for *j* = 1, …, 10. Such a choice of ***X***_*i*_ and ***β*** indicates that the three most prevalent variants are associated with the relapse. We generated failure time $T_{i}^{*}$ based on the all-cause hazard function *λ*_*i*_(*t*) = *λ*_*i*1_(*t*) + *λ*_*i*2_(*t*) and then determined whether the infection is a relapse or reinfection by a Bernoulli random variable with success probability equals to exp(***β***′***X***_*i*_)/{exp(*α*)+exp(***β***′***X***_*i*_)}. The right censoring time *C*_*i*_ was generated following a uniform distribution between 0 and *c*, where *c* is a constant controlling for 20% censoring. The observed time *T*_*i*_ is the minimum between $T_{i}^{*}$ and *C*_*i*_. We assume that for any *j* ⩽ *J*, there is one external covariate *W*_*ij*_ affecting the transition from *X*_*ij*_ to *Z*_*ij*_. For each *i* and *j*, we independently generate *W*_*ij*_ from a uniform distribution between 0 and 1, which is also independent of *X*_*ij*_.

If the event is reinfection, ***Z***_*i*_ was generated independently from the same distribution as ***X***_*i*_. If the event is a relapse, we generated ***Z***_*i*_ following the transition model (10). We let $q_{j21}=q_{j21}^{*}=0.9$ in the first scenario when ***Z***_*i*_ strongly associates with ***X***_*i*_, and $q_{j21}=q_{j21}^{*}=0.001$ in the second scenario when ***Z***_*i*_ weakly associates with ***X***_*i*_. The intercept *q*_*j*20_ was set to be 0.3 for both scenarios. We repeat the simulation 500 times for each combination of *n* and *J* under both scenarios. The operating characteristics of the two classifiers are reported in Table 1. Reported values are means and standard deviations over 500 simulations.

Table 1 shows that performance of the first classifier $I\left({\hat{\xi }}_{i}^{\left(0\right)}>0.5\right)$ is similar under both scenarios in terms of sensitivity, specificity, and overall accuracy. This result is reasonable since we only included baseline covariates and time to event information when constructing the first classifier. This information was generated using the same mechanisms under both scenarios. The second classifier $I\left({\hat{\xi }}_{i2}^{\left(1\right)}>0.5\right)$ has a better performance than the first classifier $I\left({\hat{\xi }}_{i2}^{\left(0\right)}>0.5\right)$ in scenario 1, where sensitivity, specificity, and overall accuracy are all in favor of the second classifier. The classification accuracy gets better when the sample size is larger. In scenario 1, the strong association between ***Z***_*i*_ and ***X***_*i*_ makes the transition likelihood much more informative. Therefore, the improvement in the classification performance is obvious in this scenario. However, in scenario 2, the association between ***Z***_*i*_ and ***X***_*i*_ is relatively weak. The transition likelihood contains less information in this scenario. Hence, the second classifier improves little upon the first classifier, averaging merely 12%–18% improvement in the overall accuracy, even when the sample size is larger.

When *n* and *J* are fixed, we can see that differences in the baseline hazard function *λ*_0_(*t*) barely affect the performance of both classifiers. This result is reasonable since the baseline hazard *λ*_0_(*t*) is canceled in (13). As long as the proportional hazards assumption stands, the classification accuracy is similar regardless of the true form of the baseline hazard *λ*_0_(*t*).

For high-dimensional settings, we set *α* to be 0, the first 10 components of regression coefficients in ***β*** to be log(1.5), and the rest to be 0. The remaining set-up was the same as in the low-dimensional setting. We repeat the simulation 500 times for each combination of (*n*, *J*) under two scenarios. The performance of the two classifiers is reported in Table 2.

In Table 2, we can see similar results as in Table 1. The first classifier behaves similarly under both scenarios. In scenario 1, the second classifier has perfect sensitivity and nearly perfect specificity. In scenario 2, the second classifier has similar overall accuracy as the first classifier, with slightly lower sensitivity and slightly higher specificity. The choice of the baseline hazard function *λ*_0_(*t*) barely affects the performance.

We also evaluated the accuracy of coefficient estimates $\hat{\theta }={\left(\hat{\alpha },{\hat{\beta }}^{\prime }\right)}^{\prime }$ for the high-dimensional settings, where the bias of $\hat{\alpha }$ and variable selection performance of $\hat{\beta }$ are reported in Table 2. Since we did not use transition likelihoods when estimating ***θ***, the accuracy of $\hat{\theta }$ is similar under both scenarios. The baseline hazard function was canceled when calculating the partial likelihood function (5). Therefore, it has little influence on the performance of $\hat{\theta }$ One can see that as *J* gets larger, the bias of $\hat{\alpha }$ increases. However, the performance of $\hat{\beta }$ improves since more variables are selected correctly.

In addition, we compare our classifiers with the classifiers proposed by Lin et al. (2020). We note that Lin et al. (2020) also proposed two classifiers. The first one only uses baseline covariates and the second one uses both baseline and recurrence covariates. However, they do not use time-to-event information. These classifiers require prior knowledge of the reinfection rate, which significantly affects the classification accuracy. The simulation results are provided in Section S1.1 of the Supplementary Materials.

### 5.2 Normally Distributed Covariates

In addition, we simulate for normally distributed ***X***_*i*_ and ***Z***_*i*_. For both low- and high-dimensional settings, we consider the same set-up for *α* and ***β*** as in the simulation study for binary covariates. We generated ***X***_*i*_ and ***W***_*i*_ independently from a standard normal distribution. The event time $T_{i}^{*}$, censoring time *C*_*i*_, and observed time *T*_*i*_ were all generated with the same strategy as for the binary covariates. We generated ***Z***_*i*_ based on the event type, following the transition model (11). We let $q_{j21}=q_{j21}^{*}=0.9$ in scenario 1, where ***Z***_*i*_ strongly associates with ***X***_*i*_, and let $q_{j21}=q_{j21}^{*}=0.001$ in scenario 2, where ***Z***_*i*_ weakly associates with ***X***_*i*_. We let *q*_*j*20_ = 0.3 and *ψ*_*jk*_ = 1 for each *j* under both scenarios. We repeated the simulation 500 times for each combination of *n* and *J* under both scenarios. The performance of two classifiers is reported in Tables 3 and 4 for low- and high-dimensional settings, respectively. We also reported the estimation accuracy and variable selection performance of $\hat{\theta }$ in the high-dimensional settings in Table 4.

In Table 3, the first classifier performs similarly under both scenarios. The second classifier has better performance than the first classifier under scenario 1 but comparable performance under scenario 2. Also, the change of the baseline hazard function *λ*_0_(*t*) barely affects the performance of both classifiers. A similar pattern is also observed in Table 4 in high-dimensional settings. As for $\hat{\theta }$, it has similar accuracy with various baseline hazard functions *λ*_0_(*t*). However, when *J* gets larger, the bias of $\hat{\alpha }$ increases a little, but the performance of $\hat{\beta }$ gets better. In summary, our classifiers perform similarly for both binary and normally distributed covariates.

### 5.3 Misspecified Hazard Functions

To evaluate how our classifiers perform when the hazard models in (3) and (4) are misspecified, we choose the cause-specific hazard functions as *λ*_*i*1_(*t*) = *λ*_0_(*t*) + exp(*α*) and *λ*_*i*2_(*t*) = *λ*_0_(*t*) + exp(***β***′***X***_*i*_), where *λ*_0_(*t*) = exp(*τ*), where *τ* = −0.5, 0, 0.5. In this way, the hazards are no longer proportional. We still consider both binary and normally distributed covariates and set all other parameters the transition functions the same as above. We repeat the same simulation studies for these additive hazard models. The simulation results are shown in Section S1.2 of the Supplementary Materials. We find that the first classifier does not perform well due to the misspecification of the hazard model. However, after incorporating the transition likelihood, the second classifier can still improve the classification accuracy.

## 6 *Plasmodium vivax* Malaria Infection Study

### 6.1 Identify the Cause of Recurrence Infections

As discussed in the introduction, it is essential to identify the cause of infection in *P. vivax* malaria research when the primary interest is treatment efficacy or effectiveness. In this section, we apply our proposed classifier to the *P. vivax* malaria data described in Section 1.1. We aim to classify the recurrent infection as either reinfection (*ϵ*_*i*_ = 1) or relapse (*ϵ*_*i*_ = 2). We first fit the cause-specific hazards model (3) and (4) with ***X***_*i*_ as a vector of binary covariates that indicate whether a haplotype (genetic variant) is present or absent. Parameters ***θ*** = (*α*, ***β***′)′ were estimated via the penalized partial likelihood function (5) with an *L*_1_-penalty. To choose the optimal tuning parameter *ν*, we performed a grid search in the interval [0, 3.5] and calculated the corresponding Bayesian Information Criterion (BIC) values. The BIC curve is provided in the Supplementary Materials.

We report the classification results based on *ν* = 2.05, where the BIC attains its minimum. In this case, two haplotypes (CAM.00 and CAM.04) were selected, with the proportional baseline coefficient $\text{exp}(\hat{\alpha })=0.686$. We also performed a sensitivity analysis by choosing *ν* = 0.8, where the BIC curve begins flatting out. In this case, 12 haplotypes (CAM.00, CAM.02 to CAM.10, CAM.12 and CAM.24) were selected with $\text{exp}(\hat{\alpha })=0.859$. The classification results based on *ν* = 0.8 are reported in the Supplementary Materials.

After we obtained $\hat{\theta }$, probabilities ${\hat{\xi }}_{i1}^{\left(0\right)}$ and ${\hat{\xi }}_{i2}^{\left(0\right)}$ were calculated based on formulae (6) and (7), respectively. For subjects with a recurrent infection, reading frequency for each haplotype presented at the baseline sequencing of the initial infection is used as the external covariate ***W***_*i*_. Here, covariates ***X***_*i*_ and ***Z***_*i*_ are binary variables. When the recurrent infection is reinfection (*ϵ*_*i*_ = 1), we assume ***Z***_*i*_ is independent of ***X***_*i*_ and ***W***_*i*_, but follows the same distribution as ***X***_*i*_. In this case, *ϕ*_*i*_(1) can be estimated independently without using the pseudo-likelihood function (8), and the distribution of ***Z***_*i*_ can be estimated using ***X***_*i*_ alone.

To be specific, for *ϵ*_*i*_ = 1, the transition likelihood function *ϕ*_*i*_(1, ***γ***_1_) can be written as

$$\phi_{i}\left(1,\gamma_{1}\right)=f\left(z_{i}|\epsilon_{i}=1,dN_{i}(t)=1,X_{i}=x_{i},W_{i}=w_{i}\right)=\prod_{j=1}^{J}p_{j}^{z_{ij}}{\left(1-p_{j}\right)}^{1-z_{ij}},$$

where *p*_*j*_ = *P*(*X*_*ij*_ = 1), ***γ***_1_ = (*p*_1_, …, *p*_*J*_)′. The parameter *p*_*j*_ can be consistently estimated by the sample mean ${\hat{p}}_{j}=n^{-1}\sum_{i=1}^{n}x_{ij}$. Accordingly, the transition likelihood of reinfection can be estimated by $\phi_{i}\left(1,{\hat{\gamma }}_{1}\right)=\prod_{j=1}^{J}{\hat{p}}_{j}^{z_{ij}}{\left(1-{\hat{p}}_{j}\right)}^{1-z_{ij}}$.

For *ϵ*_*i*_ = 2, when the recurrent infection is a relapse, we assume the transition likelihood follows the form of (10), that $\text{logit}\left(\mu_{ij2}\right)=q_{j20}+x_{ij}q_{j21}+w_{ij}x_{ij}q_{j21}^{*}$, with *w*_*ij*_ being the reading frequency of the *j*th haplotype of subject *i* when the haplotype is presented at the baseline sequencing, i.e., *x*_*ij*_ = 1. For computational simplicity, we assume that all haplotypes follow the same transition model, i.e., *q*_*j*20_ = *q*_0_, *q*_*j*21_ = *q*_1_, and $q_{j21}^{*}=q^{*}$ for all *j*. Then, we have $\phi_{i}\left(2,\gamma_{2}\right)=f\left(z_{i}|\epsilon_{i}=2,dN_{i}(t)=1,X_{i}=x_{i},W_{i}=w_{i}\right)=\prod_{j=1}^{J}\mu_{ij2}^{z_{ij}}{\left(1-\mu_{ij2}\right)}^{1-z_{ij}}$, where $\mu_{ij2}=\text{exp}\left(q_{0}+x_{ij}q_{1}+w_{ij}x_{ij}q^{*}\right)/\left\{1+\text{exp}\left(q_{0}+x_{ij}q_{1}+w_{ij}x_{ij}q^{*}\right)\right\}$ and ***γ***_2_ = (*q*_0_, *q*_1_, q*)′.

We replaced *ϕ*_*i*_(1, ***γ***_1_) in (8) by $\phi_{i}\left(1,{\hat{\gamma }}_{1}\right)$ and maximized the pseudo-likelihood function to obtain ${\hat{\gamma }}_{2}$. When using *ν* = 2.05, we have ${\hat{q}}_{0}=-1.366$, ${\hat{q}}_{1}=2.738$, and ${\hat{q}}^{*}=4.317$. When the recurrent infection is relapse, the parameter *q*_0_ is the log odds of a subject whose baseline sequencing did not contain haplotype *j* (*x*_*ij*_ = 0) but the follow-up sequencing at the recurrence did (*z*_*ij*_ = 1). The estimate ${\hat{q}}_{0}=-1.366$ can be transformed into an estimated transition probability of 0.203, meaning there is 20% chance that the unseen haplotype at the baseline may show up at the recurrence when the cause is relapse. Since ${\hat{q}}_{0}+{\hat{q}}_{1}=1.372$, it shows that there is around 80% chance of observing a haplotype again at the recurrence (*z*_*ij*_ = 1) when the cause is relapse and the haplotype appeared at the baseline (*x*_*ij*_ = 1). Since ${\hat{q}}^{*}=4.317$, it indicates that there is more than 99% chance of observing the same haplotype again at the recurrence (*z*_*ij*_ = 1) when the reading frequency of the haplotype is more than 80% at the baseline (*w*_*ij*_ = 0.8). When using *ν* = 0.8, we have ${\hat{q}}_{0}=-1.323$, ${\hat{q}}_{1}=2.506$ and ${\hat{q}}^{*}=4.284$. These estimates are similar to those when using *ν* = 2.05 and can be interpreted analogously.

Finally, we calculate ${\hat{\xi }}_{ik}^{\left(1\right)}$ by (9) for *k* = 1, 2 and classify the recurrent event as relapse if ${\hat{\xi }}_{i2}^{\left(1\right)}>{\hat{\xi }}_{i1}^{\left(1\right)}$ and reinfection otherwise. Table 5 contains the classification results for the 23 subjects with recurrent infection based on our proposed method using *ν* = 2.05. The tables include days to recurrence, baseline and recurrence haplotypes, the estimates $\hat{\beta }$, recurrence haplotype prevalence, two classification probabilities, and classification results from Lin et al. (2020), which analyzed the same data without utilizing the time to event information and external covariates in the estimation of transition likelihoods.

Our proposed method classifies 3 out of 23 recurrence pairs differently from Lin et al. (2020). The first pair is 87 → 87R, which was classified as relapse by Lin et al. (2020) but as reinfection by our classifier. Five variants showed up at the baseline sequencing, of which only CAM.00, the haplotype with the highest prevalence, showed up again in the recurrence sequencing. Also, the days to recurrence for this pair are 81 days, which is a relatively long time for relapse, suggesting that this recurrence event is more likely to be reinfection. The second pair is 123 → 123R, which was classified as reinfection by Lin et al. (2020) but as relapse by our classifier. Two haplotypes (CAM.00 and CAM.02) were observed at the baseline sequencing, and haplotype CAM.00 showed up again at the recurrence sequencing with CAM.01. Since only two haplotypes appeared at the recurrence, and CAM.00 is the most prevalent variant, the recurrent infection looks more likely to be a reinfection if not taking time to recurrent into consideration. However, the recurrent infection occurred only 26 days after the initial infection, which is a relatively short time compared to other reinfection cases. The only case classified as reinfection with a recurrent time less than 26 days was pair 160 → 160 R, with only 17 days to recurrence, but this is reasonable since there is no overlap between the baseline and recurrence variants. Notably, the pair 123 → 123R has 96% CAM.00 in the reading frequency at baseline, which supports the classification as relapse due to a high likelihood of observing the same variant in relapse if the variant has a high reading frequency at baseline, as suggested by large ${\hat{q}}^{*}$. The last disparity comes from pair 153 → 153R, which was classified as relapse by Lin et al. (2020) but as reinfection by our classifier. There is no overlap between initial and recurrence variants. The time to recurrence is 115 days, which is longer than any case that was classified as relapse. The only case with days to recurrence longer than this pair is pair 151 → 151R, which was classified as reinfection by both Lin et al. (2020) and our classifier. Therefore, it is more reasonable to classify pair 153 → 153R as reinfection. Overall, by considering the time to event and baseline haplotype reading frequency, our classifier achieves more consensus in this study.

### 6.2 Model Diagnosis and Sensitivity Analysis

**Figure 3:**
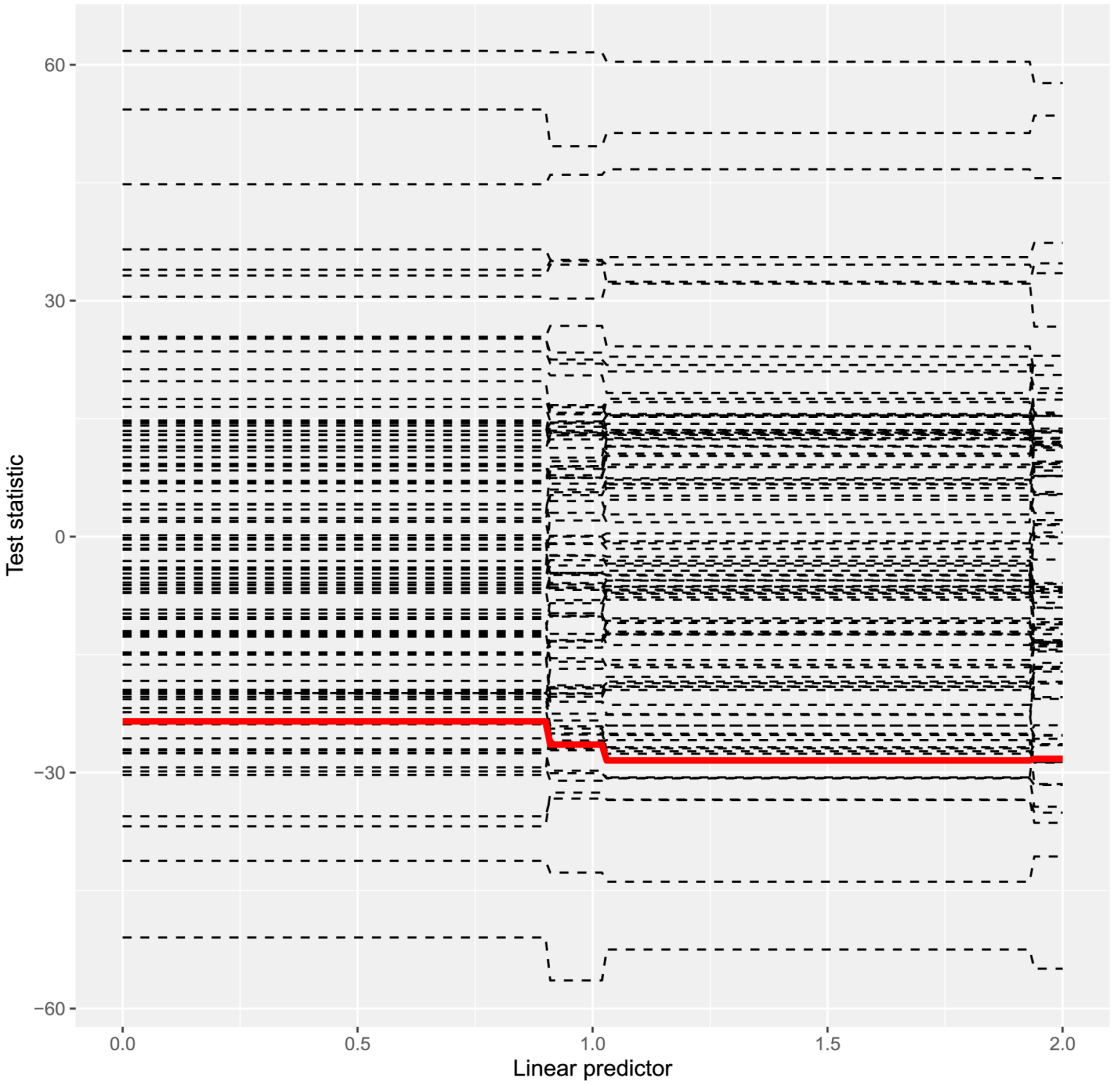
Goodness-of-fit model diagnosis for the *P. vivax* malaria data using *ν* = 2.05.

In this specific study, we assume that the cause-specific hazard functions are proportional to a baseline hazard function. Next, We verify such an assumption for the *P. vivax* malaria data using the martingale residuals method proposed in Section 2. We carry out the model diagnosis as follows. For a sequence of *x* in the range of the linear predictor ${\hat{\beta }}^{\prime }X_{i}$, we calculate the test statistic $T(x)=\sum_{i=1}^{n}I\left({\hat{\beta }}^{\prime }X_{i}⩽x\right){\hat{M}}_{i}$, where ${\hat{M}}_{i}$ is the martingale residual defined in Section 2. Using a Monte-Carlo simulation with *Q*_*i*_(*i* = 1, …, *n*) sampled independently from the standard normal distribution, the confidence band for *T*(*x*) can be constructed by calculating $T_{Q}(x)=\sum_{i=1}^{n}I\left({\hat{\beta }}^{\prime }X_{i}⩽x\right){\hat{M}}_{i}Q_{i}$. We simulate the process of *T*(*x*) by repeating the sampling. Using *ν* = 2.05, the linear predictor ${\hat{\beta }}^{\prime }X_{i}$ ranges from 0 to 1.94. Figure 3 shows the result with observed *T*(*x*) (thick solid line) and 100 simulated curves (dashed lines) for *x* ∈ [0, 1.94]. The test statistics are point-wisely within the simulated processes, with no significant indication of model violation. The model diagnosis result for the sensitivity analysis when *ν* = 0.8 is provided in the Supplementary Materials. Similarly, there is no significant model violation when using *ν* = 0.8 as well.

Misidentification of unique haplotypes is a concern in the current analysis. Low-frequency minority genetic variants that only differ in sequence by one nucleotide base pair to common variants may represent false haplotypes generated by sequencing error. We adjusted the stringency of criteria used for calling haplotypes to “collapse” such variants together, reducing the total number of 67 unique haplotypes to 32 (Hathaway et al., 2018). As a sensitivity analysis, we also analyzed the data with this total number of 32 haplotypes, based on collapsing variants with 1-nucleotide apart within the same isolate. The classification result has several disparities with the one using 67 haplotypes but mostly agreed with the one based on the method in Lin et al. (2020) using 32 haplotypes. It is not surprising to find the classification result sensitive to the identification of haplotype since our method relies on the modeling of the transition between variants. The collapse of variants and corresponding classification results using 32 haplotypes are provided in the Supplementary Materials.

## 7 Discussion

We proposed a classification method for identifying the latent cause of events under competing risks set-up, which utilizes both time to event and transition likelihood information for better classification performance. By considering the transition likelihood, we utilize more information when constructing the classifier, which leads to better performance than the classifier using only baseline information. The method can be applied regardless of the true form of the baseline hazard function, and can also be applied to a variety of covariate data types. We examined the performance of our method through simulation studies under various settings as well as real data analysis, which shows high reliability of our method.

When modeling the outcomes of competing risks, we assumed a proportional hazards model with a common baseline hazard function for every cause-specific hazard. When the hazards share the same covariates, the model may not be identifiable. To avoid the identifiability issue when analyzing the *P. vivax* malaria data, we assume the reinfection process is independent of any baseline covariates in ***X***_*i*_ but has a hazard function proportional to a baseline hazard *λ*_0_(*t*). This assumption is reasonable for our data but may not be ideal for a general case. Alternative approaches for the estimation of the hazard functions call for more investigation. In our current approach, we assume the transition of covariates is independent of time. It will be of interest to generalize the transition model to be a function of time. A possible approach is to include time *t*_*i*_ as a covariate in the model for *μ*_*ijk*_. This approach is somehow restricted to a linear function of time, which is subject to model misspecification.

The statistical inference of regression coefficients ***β*** is also a topic worth investigating. While the current method can perform variable selection on ***β*** with high accuracy, inferring the significance of these selected variables needs more work. We also remark that if one would like to evaluate the treatment efficacy using our approach, they can include treatment as a covariate in (4). Then, using the same penalized partial likelihood method as shown in (14), they can estimate the coefficient corresponding to the treatment for the treatment efficacy.

Finally, we point out that due to the nature of the *P. vivax* malaria, causes for recurrence are often unobservable. This problem motivates us to develop the classification method for totally unobservable causes. For other applications, when causes may be partially observed, one can plug their cause-specific hazards into the partial likelihood function (1) for subjects with observed causes and treat the rest causes as missing data. Then, EM algorithms may be utilized to obtain $\hat{\theta }$, based on which one can still build the two proposed classifiers. It will be of great interest to study the estimator’s efficiency improvement by the transition likelihood in the future study.

## Supplementary Material

jds1026_s001

## Figures and Tables

**Table 1:** Classification performance of proposed classifiers with low-dimensional binary covariates.

			$I\left({\hat{\xi }}_{i}^{\left(0\right)}>0.5\right)$			$I\left({\hat{\xi }}_{i}^{\left(1\right)}>0.5\right)$		
Scenario	*τ*	(*n, J*)	Sensitivity	Specificity	Overall	Sensitivity	Specificity	Overall
1	−0.5	(400, 10)	50.3 (20.2)	59.0 (19.2)	53.6 (5.1)	90.7 (4.2)	94.3 (3.2)	92.1 (2.2)
		(800, 20)	50.1 (19.9)	59.6 (19.2)	54.0 (4.5)	97.8 (0.9)	98.7 (0.8)	98.0 (0.5)
	0	(400, 10)	49.1 (18.4)	60.1 (17.6)	53.6 (4.6)	89.3 (10.8)	93.2 (10.8)	90.9 (10.4)
		(800, 20)	49.6 (18.2)	59.7 (17.8)	53.8 (3.9)	97.9 (0.9)	98.2 (0.8)	98.0 (0.6)
	0.5	(400, 10)	48.3 (18.7)	61.9 (17.3)	53.9 (4.8)	88.9 (12.2)	92.4 (12.0)	90.3 (11.8)
		(800, 20)	50.2 (17.8)	59.5 (17.2)	54.1 (3.9)	97.9 (0.8)	98.1 (0.8)	98.0 (0.5)
2	−0.5	(400, 10)	48.7 (19.7)	60.6 (18.8)	53.6 (5.0)	66.3 (16.9)	72.5 (30.2)	68.8 (21.2)
		(800, 20)	50.7 (18.7)	58.8 (17.9)	54.0 (4.1)	66.2 (14.6)	71.9 (13.4)	68.6 (11.9)
	0	(400, 10)	49.3 (19.7)	59.6 (18.5)	53.6 (5.1)	64.4 (18.2)	69.2 (32.3)	66.3 (23.1)
		(800, 20)	51.6 (17.9)	58.5 (17.4)	54.5 (3.9)	66.2 (14.7)	72.1 (13.3)	68.6 (11.9)
	0.5	(400, 10)	49.2 (18.6)	60.6 (17.6)	53.7 (4.5)	68.7 (16.6)	74.9 (27.5)	71.1 (20.4)
		(800, 20)	50.8 (18.1)	58.8 (17.5)	54.0 (4.1)	66.3 (14.5)	72.3 (12.9)	68.7 (11.8)

Sensitivity, specificity and overall accuracy are given as percentages.

Reported values are means and standard deviations over 500 simulations.

**Table 2:** Classification and variable selection performance of proposed classifiers with high-dimensional binary covariates.

			$I\left({\hat{\xi }}_{i}^{\left(0\right)}>0.5\right)$			$I\left({\hat{\xi }}_{i}^{\left(1\right)}>0.5\right)$			$\hat{\alpha }$	$\hat{\beta }$		
Scenario	*τ*	(*n, J*)	Sensitivity	Specificity	Overall	Sensitivity	Specificity	Overall	Bias	Sensitivity	Specificity	Overall
1	−0.5	(100, 200)	98.1 (3.0)	4.4 (6.8)	76.1 (4.6)	100 (0)	96.3 (6.0)	99.5 (0.7)	0.48 (0.05)	75.2 (12.8)	57.1 (2.0)	58.0 (2.1)
		(200, 400)	96.7 (3.5)	8.4 (7.1)	75.3 (3.1)	100 (0)	100 (0)	100 (0)	0.51 (0.01)	87.2 (10.6)	65.7 (1.3)	66.7 (1.4)
	0	(100, 200)	97.8 (3.4)	5.3 (7.7)	74.8 (4.5)	100 (0)	97.4 (4.5)	99.6 (0.6)	0.49 (0.04)	72.9 (13.0)	58.2 (2.1)	59.0 (2.2)
		(200, 400)	95.7 (3.7)	9.2 (7.5)	75.1 (3.5)	100 (0)	100 (0)	100 (0)	0.51 (0.01)	87.0 (10.6)	65.7 (1.6)	65.9 (1.4)
	0.5	(100, 200)	97.6 (2.8)	4.6 (6.8)	75.3 (4.5)	100 (0)	96.7 (6.0)	99.5 (0.7)	0.49 (0.04)	72.9 (13.6)	58.0 (2.3)	58.7 (2.4)
		(200, 400)	95.8 (3.8)	9.8 (7.1)	75.0 (3.3)	100 (0)	99.9 (0.8)	100 (0)	0.51 (0.02)	87.1 (11.2)	65.4 (1.4)	66.0 (1.5)
2	−0.5	(100, 200)	97.9 (2.9)	4.9 (5.8)	75.8 (4.6)	91.8 (4.5)	13.0 (8.2)	73.1 (5.0)	0.49 (0.05)	78.3 (14.3)	62.2 (2.5)	61.1 (2.3)
		(200, 400)	96.2 (3.8)	8.8 (7.9)	75.3 (3.3)	90.7 (5.6)	14.9 (9.1)	72.7 (4.0)	0.50 (0.02)	73.8 (14.1)	67.2 (1.7)	66.3 (1.7)
	0	(100, 200)	97.5 (3.1)	6.4 (6.9)	74.9 (4.3)	91.9 (4.8)	14.3 (9.2)	72.7 (4.2)	0.50 (0.04)	79.2 (15.8)	62.6 (2.2)	61.4 (2.4)
		(200, 400)	95.8 (3.8)	8.8 (7.4)	74.8 (3.5)	90.6 (5.1)	15.4 (8.3)	72.5 (3.9)	0.51 (0.02)	75.3 (14.5)	67.5 (1.9)	66.5 (1.4)
	0.5	(100, 200)	97.4 (2.6)	5.7 (6.1)	75.4 (4.4)	91.5 (4.8)	13.6 (8.7)	72.8 (4.8)	0.51 (0.04)	79.0 (15.8)	61.6 (2.3)	60.5 (2.3)
		(200, 400)	95.6 (3.7)	9.5 (7.7)	74.7 (3.1)	90.3 (5.5)	16.3 (8.3)	72.2 (3.8)	0.51 (0.02)	73.1 (14.9)	66.2 (1.5)	65.4 (1.5)

Sensitivity, specificity and overall accuracy are given as percentages.

Reported values are means and standard deviations over 500 simulations.

**Table 3:** Classification of proposed classifiers with low-dimensional continuous covariates.

			$I\left({\hat{\xi }}_{i}^{\left(0\right)}>0.5\right)$			$I\left({\hat{\xi }}_{i}^{\left(1\right)}>0.5\right)$		
Scenario	*τ*	(*n, J*)	Sensitivity	Specificity	Overall	Sensitivity	Specificity	Overall
1	−0.5	(400, 10)	67.8 (4.1)	54.8 (4.5)	61.6 (3.2)	97.5 (1.4)	97.5 (1.6)	97.6 (1.0)
		(800, 20)	64.9 (2.8)	58.6 (2.5)	61.8 (1.9)	99.8 (0.2)	99.8 (0.3)	99.7 (0.1)
	0	(400, 10)	65.9 (4.4)	57.1 (4.3)	61.6 (3.1)	97.6 (1.3)	97.5 (1.3)	97.5 (0.9)
		(800, 20)	63.6 (2.4)	59.5 (2.6)	61.6 (1.9)	99.7 (0.3)	99.7 (0.3)	99.7 (0.2)
	0.5	(400, 10)	64.7 (3.5)	60.2 (3.9)	62.4 (3.0)	97.6 (1.2)	97.4 (1.1)	97.5 (0.7)
		(800, 20)	62.5 (2.5)	60.4 (2.2)	61.5 (1.7)	99.7 (0.3)	99.7 (0.3)	99.7 (0.2)
2	−0.5	(400, 10)	67.6 (4.3)	54.9 (4.3)	61.5 (3.1)	68.5 (4.3)	56.2 (4.8)	62.5 (3.1)
		(800, 20)	64.6 (2.7)	58.4 (2.8)	61.8 (2.5)	67.9 (2.4)	62.7 (3.8)	65.4 (2.0)
	0	(400, 10)	65.7 (3.9)	57.4 (4.2)	61.7 (3.0)	67.0 (3.9)	59.1 (4.4)	63.1 (3.0)
		(800, 20)	63.6 (2.6)	59.9 (2.7)	61.8 (1.8)	67.3 (2.4)	64.0 (2.6)	65.6 (1.8)
	0.5	(400, 10)	63.9 (3.6)	59.6 (4.0)	61.8 (2.7)	65.5 (3.2)	61.1 (4.0)	63.5 (2.5)
		(800, 20)	62.8 (2.6)	60.6 (2.5)	61.7 (1.8)	66.4 (2.5)	64.6 (2.6)	65.5 (1.7)

Sensitivity, specificity and overall accuracy are given as percentages.

Reported values are means and standard deviations over 500 simulations.

**Table 4:** Classification performance of proposed classifiers with high-dimensional continuous covariates.

Scenario	*τ*	(*n, J*)	$I\left({\hat{\xi }}_{i}^{\left(0\right)}>0.5\right)$			$I\left({\hat{\xi }}_{i}^{\left(1\right)}>0.5\right)$			$\hat{\alpha }$	$\hat{\beta }$		
			Sensitivity	Specificity	Overall	Sensitivity	Specificity	Overall	Bias	Sensitivity	Specificity	Overall
1	−0.5	(100, 200)	85.8 (5.8)	29.8 (8.7)	59.2 (5.5)	98.7 (11.4)	99.7 (5.7)	99.5 (6.7)	0.44 (0.02)	69.5 (14.8)	57.3 (2.3)	58.5 (2.9)
		(200, 400)	88.7 (3.5)	27.1 (5.9)	60.0 (4.1)	100 (0)	100 (0)	100 (0)	0.47 (0.01)	82.0 (11.9)	60.4 (1.9)	60.9 (1.9)
	0	(100, 200)	83.4 (5.2)	33.6 (7.4)	59.0 (5.4)	99.0 (10.5)	99.6 (5.7)	99.1 (7.2)	0.45 (0.02)	70.8 (15.4)	57.3 (3.0)	57.9 (3.0)
		(200, 400)	85.2 (4.5)	31.9 (5.6)	59.6 (3.9)	100 (0)	100 (0)	100 (0)	0.47 (0.01)	82.7 (12.5)	59.3 (1.9)	59.9 (1.9)
	0.5	(100, 200)	81.9 (5.3)	37.5 (7.2)	60.1 (5.2)	98.3 (12.8)	99.7 (5.8)	99.0 (8.1)	0.44 (0.02)	71.4 (14.5)	56.1 (2.7)	56.9 (2.9)
		(200, 400)	84.5 (3.7)	34.0 (5.1)	59.6 (3.9)	100 (0)	100 (0)	100 (0)	0.47 (0.01)	85.0 (11.0)	58.6 (1.7)	59.2 (1.6)
2	−0.5	(100, 200)	85.5 (5.4)	29.3 (7.9)	58.9 (5.7)	94.2 (3.6)	23.4 (7.9)	60.8 (6.3)	0.43 (0.02)	62.3 (15.4)	64.8 (2.8)	64.6 (2.9)
		(200, 400)	84.0 (4.3)	32.0 (5.8)	59.5 (4.1)	96.3 (2.2)	31.7 (6.9)	65.8 (4.7)	0.47 (0.01)	75.6 (14.4)	68.0 (1.8)	68.2 (1.9)
	0	(100, 200)	82.9 (6.0)	34.2 (7.2)	59.5 (5.4)	92.9 (4.0)	27.7 (7.6)	61.7 (5.6)	0.44 (0.02)	64.8 (15.6)	64.1 (2.7)	64.1 (2.9)
		(200, 400)	81.3 (4.2)	36.5 (5.9)	59.6 (3.9)	95.7 (2.2)	35.7 (6.7)	66.5 (4.7)	0.47 (0.01)	76.8 (14.1)	67.4 (2.0)	67.7 (2.0)
	0.5	(100, 200)	82.0 (5.7)	37.8 (7.1)	60.1 (5.5)	92.5 (4.1)	31.0 (8.1)	62.1 (6.1)	0.45 (0.02)	63.8 (15.9)	63.7 (2.8)	63.7 (2.9)
		(200, 400)	79.9 (4.0)	38.5 (5.5)	59.5 (3.7)	95.1 (2.3)	37.8 (6.6)	66.9 (4.5)	0.46 (0.01)	77.1 (13.6)	66.7 (2.1)	67.4 (2.1)

Sensitivity, specificity and overall accuracy are given as percentages.

Reported values are means and standard deviations over 500 simulations.

**Table 5:** Classification of the first recurrent infection (*ν* = 2.05).

Recurrence Pair	Days to Recurrence	Baseline Variants	$\hat{\beta }$	${\hat{\xi }}^{\left(0\right)}$	Recurrence Variants	Variant Prevalence	${\hat{\xi }}^{\left(1\right)}$	Class by our method	Class by Lin et al.
10 → 10R	84	CAM.00	0.907	0.783	CAM.00	0.590	0.995	Relapse	Relapse
		CAM.11	0		CAM.11	0.077			
					CAM.15	0.013			
31 → 31R	84	CAM.00	0.907	0.910	CAM.16	0.006	0.988	Relapse	Relapse
		CAM.02	0						
		CAM.04	1.026						
		CAM.31	0						
36 → 36R	99	CAM.00	0.907	0.910	CAM.01	0.269	0.645	Relapse	Relapse
		CAM.01	0		CAM.02	0.41			
		CAM.02	0		CAM.07	0.192			
		CAM.03	0		CAM.17	0.064			
		CAM.04	1.026						
		CAM.05	0						
		CAM.06	0						
		CAM.07	0						
		CAM.09	0						
		CAM.11	0						
68 → 68R	99	CAM.00	0.907	0.910	CAM.10	0.077	0.997	Relapse	Relapse
		CAM.02	0						
		CAM.04	1.026						
		CAM.10	0						
80 → 80R	56	CAM.00	0.907	0.910	CAM.00	0.590	0.000	Reinfection	Reinfection
		CAM.04	1.026		CAM.01	0.269			
		CAM.05	0		CAM.02	0.410			
		CAM.08	0		CAM.03	0.295			
		CAM.09	0		CAM.05	0.231			
		CAM.24	0		CAM.06	0.231			
		CAM.27	0		CAM.07	0.192			
					CAM.08	0.154			
					CAM.12	0.064			
					CAM.41	0.013			
81 → 81R	35	CAM.00	0.907	0.783	CAM.00	0.590	0.974	Relapse	Relapse
		CAM.01	0		CAM.01	0.269			
		CAM.51	0						
82 → 82R	56	CAM.00	0.907	0.910	CAM.00	0.590	0.674	Relapse	Relapse
		CAM.03	0		CAM.01	0.269			
		CAM.04	1.026		CAM.03	0.295			
		CAM.10	0		CAM.46	0.006			
87 → 87R	81	CAM.00	0.907	0.783	CAM.00	0.590	0.424	Reinfection	Relapse
		CAM.01	0		CAM.07	0.192			
		CAM.02	0		CAM.08	0.154			
		CAM.08	0		CAM.53	0.013			
		CAM.24	0						
89 → 89R	14	CAM.00	0.907	0.910	CAM.01	0.269	0.052	Reinfection	Reinfection
		CAM.04	1.026		CAM.09	0.077			
		CAM.06	0		CAM.20	0.026			
		CAM.08	0		CAM.27	0.038			
		CAM.10	0						
		CAM.12	0						
96 → 96R	71	CAM.00	0.907	0.910	CAM.00	0.590	0.983	Relapse	Relapse
		CAM.02	0		CAM.30	0.013			
		CAM.04	1.026						
		CAM.08	0						
112 → 112R	67	CAM.00	0.907	0.910	CAM.00	0.590	0.670	Relapse	Relapse
		CAM.01	0		CAM.01	0.269			
		CAM.02	0		CAM.02	0.410			
		CAM.04	1.026						
		CAM.07	0						
		CAM.12	0						
		CAM.40	0						
		CAM.42	0						
		CAM.60	0						
118 → 118R	89	CAM.08	0	0.593	CAM.01	0.269	0.008	Reinfection	Reinfection
					CAM.02	0.410			
					CAM.25	0.006			
					CAM.39	0.006			
123 → 123R	26	CAM.00	0.907	0.783	CAM.00	0.590	0.700	Relapse	Reinfection
		CAM.02	0		CAM.01	0.269			
125 → 125R	82	CAM.02	0	0.593	CAM.00	0.590	0.000	Reinfection	Reinfection
					CAM.01	0.269			
					CAM.02	0.410			
					CAM.04	0.346			
					CAM.09	0.077			
					CAM.13	0.006			
					CAM.14	0.026			
					CAM.38	0.006			
					CAM.45	0.006			
126 → 126R	85	CAM.00	0.907	0.910	CAM.01	0.269	0.975	Relapse	Relapse
		CAM.01	0		CAM.07	0.192			
		CAM.02	0		CAM.33	0.006			
		CAM.03	0						
		CAM.04	1.026						
		CAM.05	0						
		CAM.06	0						
		CAM.07	0						
		CAM.22	0						
		CAM.50	0						
130 → 130R	68	CAM.00	0.907	0.910	CAM.00	0.590	0.997	Relapse	Relapse
		CAM.02	0		CAM.04	0.346			
		CAM.03	0		CAM.12	0.064			
		CAM.04	1.026						
		CAM.12	0						
151 → 151R	126	CAM.03	0	0.593	CAM.00	0.590	0.325	Reinfection	Reinfection
		CAM.05	0		CAM.08	0.154			
		CAM.08	0		CAM.14	0.026			
					CAM.64	0.006			
152 → 152R	94	CAM.00	0.907	0.783	CAM.00	0.590	0.153	Reinfection	Reinfection
		CAM.01	0		CAM.01	0.269			
					CAM.05	0.231			
					CAM.07	0.192			
153 → 153R	115	CAM.00	0.907	0.910	CAM.02	0.410	0.425	Reinfection	Relapse
		CAM.04	1.026		CAM.20	0.026			
		CAM.07	0						
		CAM.55	0						
154 → 154R	64	CAM.00	0.907	0.783	CAM.03	0.295	0.116	Reinfection	Reinfection
		CAM.06	0		CAM.05	0.231			
		CAM.57	0		CAM.06	0.231			
160 → 160R	17	CAM.02	0	0.803	CAM.00	0.590	0.000	Reinfection	Reinfection
		CAM.04	1.026		CAM.03	0.295			
		CAM.07	0		CAM.05	0.231			
					CAM.10	0.077			
					CAM.61	0.006			
177 → 177R	84	CAM.00	0.907	0.910	CAM.01	0.269	0.773	Relapse	Relapse
		CAM.04	1.026						
		CAM.07	0						
179 → 179R	84	CAM.03	0	0.593	CAM.01	0.269	0.234	Reinfection	Reinfection
		CAM.05	0		CAM.13	0.006			
		CAM.07	0						
		CAM.09	0						
		CAM.17	0						
		CAM.22	0						
