# Supplementary material for: Dynamic Classification of Plasmodium vivax Malaria Recurrence: An Application of Classifying Unknown Cause of Failure in Competing Risks
Source: J Data Sci. Author manuscript; Available in PMC 2022 Aug 3. (PMC9347664; doi:10.6339/21-jds1026)
Supplement: jds1026_s001 [file NIHMS1786249-supplement-jds1026_s001.pdf]

Journal of Data Science xx (xx), 1–20 DOI: xx.xxxx/xxxxxxxxx
xxx 20xx

Supplementary Materials for "Dynamic Classification of *Plasmodium vivax* Malaria Recurrence: An Application of Classifying Unknown Cause of Failure in Competing Risks"

Yutong Liu[1], Feng-Chang Lin[1], Jessica T. Lin[2], and Quefeng Li *[1]

[1]*Department of Biostatistics, University of North Carolina at Chapel Hill, Chapel Hill, North Carolina, U.S.A.*

[2]*Institute of Global Health and Infectious Diseases, University of North Carolina at Chapel Hill, Chapel Hill, North Carolina, U.S.A.*

S1 Additional simulation results

S1.1 Simulation results of the method by Lin et al. (2020) for binary covariates

We apply the method proposed by Lin et al. (2020) to the same data generated in Section 5.1. The classification results are given in Tables S1 and S2. Their method requires the reinfection rate to be known a priori. We set the infection rate to be 0.05, the same as Lin et al. (2020). Under the low-dimensional setting, we could see that their first classifier always classifies events to relapse, which leads to a 100% sensitivity and 0% specificity. Its poor performance is due to the fact that such a classifier does not use the time-to-event information for classification. Moreover, their second classifier's performance is still worse than our classifier. Again, this is due to not using time-to-event information. We also observe that under both scenarios, for fixed n and J as τ gets larger the classifier's sensitivity increases while specificity decreases. The overall accuracy increases when τ gets larger. Comparing the two scenarios, the specificity does not differ much but the sensitivity and overall accuracy are both higher in the scenario when the association between baseline and recurrent covariates is strong. Comparing Tables 1 and 2 with Tables S1 and S2, we can see that our classifiers perform uniformly better than those proposed in Lin et al. (2020).

S1.2 Simulation results for misspecified hazard functions

To investigate how our classifiers perform when the proportional hazard assumption is violated, we repeat the simulation studies in Section 5 with additive hazard models. For reinfection, we assume its hazard function is $\lambda_0(t) + \exp(\alpha)$. For relapse, we assume its hazard function is $\lambda_0(t) + \exp(\boldsymbol{\beta}'\boldsymbol{X}_i)$. In both cases, we choose $\lambda_0(t) = \exp(\tau)$, for $\tau = -0.5$, 0 and 0.5. We keep all other settings the same as in Section 5 and repeat simulations for both binary and normal covariates under low- and high-dimensional scenarios. Tables S3–S6 give the classification results in each scenario. Comparing the classification accuracy of the two classifiers, we find that the second classifier's overall accuracy is much better than the first one in every scenario. Also, when the association between baseline and recurrence covariates becomes larger, the improvement becomes more profound. This result suggests that considering transition likelihood benefits classification even if the time-to-event model is misspecified. On the other hand, comparing these tables with the ones in Section 5, we find that when the hazard functions are misspecified,

*quefeng@email.unc.edu

Received xx, 20xx; Accepted xx, 20xx

Table S1: Classification performance of classifiers from Lin et al. (2020) with low-dimensional binary covariates.

			$I(\widehat{\xi}_i^{(0)} > 0.5)$			$I(\widehat{\xi}_i^{(1)} > 0.5)$		
Scenario	τ	$(n,\ J)$	Sensitivity	Specificity	Overall	Sensitivity	Specificity	Overall
1	-0.5	(400, 10)	100 (0)	0 (0)	63.5 (0.5)	66.7 (1.1)	9.9 (1.8)	45.9 (0.3)
		(800, 20)	100 (0)	0 (0)	63.2 (0.1)	83.7 (0.9)	4.1 (0.1)	54.4 (0.5)
	0	(400, 10)	100 (0)	0 (0)	60.3 (0.7)	77.9 (1.1)	4.5 (0.4)	48.8 (1.0)
		(800, 20)	100 (0)	0 (0)	63.5 (0.9)	90.6 (0.4)	1.9 (0)	58.2 (1.1)
	0.5	(400, 10)	100 (0)	0 (0)	60.7 (0.2)	87.7 (1.4)	0.3 (0.9)	53.3 (0.4)
		(800, 20)	100 (0)	0 (0)	64.8 (0.4)	97.0 (0.4)	1.0 (0.2)	63.3 (0.7)
2	-0.5	(400, 10)	100 (0)	0 (0)	63.6 (0.2)	43.6 (0.6)	10.2 (0.9)	30.8 (0.1)
		(800, 20)	100 (0)	0 (0)	63.2 (0.1)	72.5 (2.2)	4.8 (0.2)	47.6 (1.2)
	0	(400, 10)	100 (0)	0 (0)	60.3 (0.7)	62.3 (0.2)	4.5 (0.4)	39.4 (0.3)
		(800, 20)	100 (0)	0 (0)	63.5 (0.9)	81.1 (1.5)	1.9 (0.0)	52.2 (1.8)
	0.5	(400, 10)	100 (0)	0 (0)	60.2 (1.4)	77.0 (3.6)	0.1 (0.2)	46.4 (3.1)
		(800, 20)	100 (0)	0 (0)	64.8 (0.4)	93.8 (0.6)	1.0 (0.2)	61.1 (0.9)

Sensitivity, specificity and overall accuracy are given as percentages.
Reported values are means and standard deviations over 500 simulations.

the classification accuracy is not as good as when the hazard functions are correctly specified. These results are not surprising as our classifiers rely on the time-to-event information to classify causes.

Table S2: Classification performance of classifiers from Lin et al. (2020) with high-dimensional binary covariates.

			$I(\widehat{\xi}_i^{(0)} > 0.5)$			$I(\widehat{\xi}_i^{(1)} > 0.5)$		
Scenario	τ	$(n,\ J)$	Sensitivity	Specificity	Overall	Sensitivity	Specificity	Overall
1	-0.5	(100, 200)	100 (0)	0 (0)	76.4 (4.5)	61.3 (45.9)	39.2 (46.5)	65.4 (24.5)
		(200, 400)	100 (0)	0 (0)	75.8 (2.9)	52.0 (50.1)	48.0 (50.1)	51.5 (25.9)
	0	(100, 200)	100 (0)	0 (0)	75.7 (4.2)	81.0 (35.0)	19.8 (36.0)	66.6 (17.7)
		(200, 400)	100 (0)	0 (0)	75.9 (2.7)	85.8 (34.8)	14.1 (34.9)	68.8 (17.9)
	0.5	(100, 200)	100 (0)	0 (0)	76.1 (4.4)	89.2 (21.5)	11.0 (22.4)	70.4 (11.6)
		(200, 400)	100 (0)	0 (0)	76.1 (2.8)	83.2 (36.8)	17.0 (36.9)	67.7 (19.1)
2	-0.5	(100, 200)	100 (0)	0 (0)	76.0 (4.9)	58.0 (49.3)	39.3 (46.9)	53.4 (26.8)
		(200, 400)	100 (0)	0 (0)	76.6 (3.3)	47.5 (49.8)	52.1 (49.9)	49.0 (26.7)
	0	(100, 200)	100 (0)	0 (0)	75.1 (4.1)	74.0 (44.1)	18.2 (32.5)	59.4 (26.0)
		(200, 400)	100 (0)	0 (0)	75.9 (2.9)	83.0 (37.7)	16.9 (37.5)	66.8 (20.0)
	0.5	(100, 200)	100 (0)	0 (0)	75.4 (4.7)	68.2 (46.8)	18.5 (28.7)	55.7 (29.0)
		(200, 400)	100 (0)	0 (0)	76.2 (2.8)	78.2 (41.4)	21.3 (40.7)	64.3 (22.3)

Sensitivity, specificity and overall accuracy are given as percentages.
Reported values are means and standard deviations over 500 simulations.

S1.3 Parameter estimation for low-dimensional binary covariates model

For two low-dimensional settings in Section 5, we represent their parameter estimation accuracy in Tables S7 and S8.

S2 Additional results of *Plasmodium vivax* malaria infection study

We here give more details about the *Plasmodium vivax* Malaria Infection study. Figure S1 shows the 23 subjects' time to first recurrence infection. Table S9 shows the classification results given by our method when using 67 haplotypes and $\nu = 0.8$. Figure S2 is the BIC curve when analyzing the *P. vivax* malaria data. Table S10 details the haplotypes that were collapsed with other haplotypes. Any other haplotypes not shown in the first column of the table were not collapsed. Table S11 shows the classification results given by our method when using 32 haplotypes, in which case the BIC attains its minimum at $\nu = 1.6$. Figure S3 shows the goodness-of-fit test result when using $\nu = 0.8$. The test statistics are point-wisely within the simulated processes, with no significant pattern of model violation.

Table S3: Classification performance of proposed classifiers with low-dimensional binary covariates when hazard models are misspecified.

			$I(\widehat{\xi}_i^{(0)} > 0.5)$			$I(\widehat{\xi}_i^{(1)} > 0.5)$		
Scenario	τ	$(n,\ J)$	Sensitivity	Specificity	Overall	Sensitivity	Specificity	Overall
1	-0.5	(400, 10)	40.6 (11.6)	45.3 (10.9)	41.4 (3.1)	84.9 (3.1)	89.9 (1.9)	87.0 (1.7)
		(800, 20)	33.1 (22.5)	48.8 (21.8)	38.8 (3.7)	91.9 (2.3)	93.5 (0.9)	92.6 (1.2)
	0	(400, 10)	35.2 (12.1)	49.3 (11.1)	40.5 (3.3)	84.5 (3.0)	90.2 (1.9)	86.9 (1.6)
		(800, 20)	28.0 (19.6)	53.6 (18.8)	38.5 (2.9)	91.6 (2.3)	93.6 (0.8)	92.5 (1.2)
	0.5	(400, 10)	31.7 (11.3)	51.9 (10.9)	40.1 (2.8)	84.2 (3.0)	90.7 (1.9)	86.8 (1.7)
		(800, 20)	27.0 (19.8)	53.9 (19.1)	38.3 (2.6)	91.1 (1.5)	93.6 (0.7)	92.1 (0.7)
2	-0.5	(400, 10)	37.9 (11.1)	48.0 (11.5)	41.0 (3.0)	43.2 (5.4)	57.7 (3.7)	49.5 (2.7)
		(800, 20)	33.5 (20.8)	48.7 (19.8)	38.8 (3.8)	49.5 (12.5)	64.0 (3.5)	55.8 (6.0)
	0	(400, 10)	34.6 (11.3)	50.4 (11.2)	40.4 (2.9)	42.8 (5.3)	57.9 (3.2)	49.4 (2.6)
		(800, 20)	30.4 (21.9)	50.9 (21.3)	38.6 (3.1)	48.5 (12.8)	64.0 (4.0)	55.5 (5.8)
	0.5	(400, 10)	32.6 (11.7)	50.9 (11.9)	40.0 (3.2)	42.4 (5.6)	58.3 (4.5)	49.0 (2.8)
		(800, 20)	28.5 (21.2)	52.0 (20.7)	38.5 (2.5)	47.8 (12.9)	64.0 (4.0)	55.2 (5.6)

Sensitivity, specificity and overall accuracy are given as percentages.
Reported values are means and standard deviations over 500 simulations.

Table S4: Classification performance of proposed classifiers with high-dimensional binary covariates when hazard models are misspecified.

			$I(\widehat{\xi}_i^{(0)} > 0.5)$			$I(\widehat{\xi}_i^{(1)} > 0.5)$		
Scenario	τ	$(n,\ J)$	Sensitivity	Specificity	Overall	Sensitivity	Specificity	Overall
1	-0.5	(100, 200)	77.1 (4.2)	1.7 (6.6)	50.9 (4.7)	92.4 (5.3)	10.0 (7.9)	69.1 (4.9)
		(200, 400)	72.1 (5.6)	10.3 (8.5)	48.8 (3.7)	86.0 (6.1)	19.9 (8.1)	66.5 (4.0)
	0	(100, 200)	76.0 (6.1)	3.4 (9.6)	47.9 (5.7)	92.3 (6.8)	11.2 (10.3)	66.8 (5.7)
		(200, 400)	72.1 (5.2)	10.3 (8.0)	47.3 (3.3)	85.8 (6.0)	20.3 (8.3)	65.2 (3.7)
	0.5	(100, 200)	76.0 (5.3)	3.9 (8.3)	45.9 (5.0)	91.2 (6.7)	12.0 (9.3)	64.5 (5.5)
		(200, 400)	69.9 (5.5)	12.9 (8.8)	44.3 (3.2)	83.1 (6.1)	22.0 (8.4)	62.0 (3.6)
2	-0.5	(100, 200)	76.6 (5.7)	2.1 (7.9)	50.1 (5.1)	77.9 (41.5)	14.2 (43.0)	52.7 (35.5)
		(200, 400)	71.2 (5.2)	11.0 (8.5)	49.7 (4.5)	71.5 (42.3)	18.9 (50.1)	50.3 (29.8)
	0	(100, 200)	77.8 (3.1)	1.3 (5.7)	47.8 (4.9)	71.2 (45.3)	4.9 (35.6)	48.1 (35.7)
		(200, 400)	69.7 (4.3)	10.2 (6.3)	45.5 (4.2)	64.8 (39.4)	7.2 (40.5)	45.4 (30.2)
	0.5	(100, 200)	75.5 (6.1)	3.4 (8.3)	45.2 (4.9)	60.2 (49.0)	9.0 (39.4)	46.2 (39.7)
		(200, 400)	66.4 (5.7)	10.8 (9.6)	43.7 (5.3)	52.1 (47.6)	17.6 (38.0)	44.7 (36.0)

Sensitivity, specificity and overall accuracy are given as percentages.
Reported values are means and standard deviations over 500 simulations.

Table S5: Classification performance of proposed classifiers with low-dimensional continuous covariates when hazard models are misspecified.

			$I(\widehat{\xi}_i^{(0)} > 0.5)$			$I(\widehat{\xi}_i^{(1)} > 0.5)$		
Scenario	τ	$(n,\ J)$	Sensitivity	Specificity	Overall	Sensitivity	Specificity	Overall
1	-0.5	(400, 10)	14.1 (4.6)	74.1 (4.1)	43.1 (2.6)	81.4 (2.4)	88.6 (0.9)	84.9 (1.2)
		(800, 20)	12.8 (3.9)	77.9 (2.6)	41.2 (1.7)	83.5 (1.7)	90.5 (1.2)	87.8 (0.8)
	0	(400, 10)	11.2 (4.1)	74.0 (4.0)	41.6 (2.4)	80.5 (2.6)	89.8 (0.9)	84.5 (1.4)
		(800, 20)	8.9 (3.2)	77.3 (3.0)	40.2 (1.9)	83.3 (2.1)	91.2 (1.1)	86.9 (0.9)
	0.5	(400, 10)	9.1 (4.3)	74.9 (3.8)	40.9 (2.6)	81.0 (2.8)	88.7 (0.9)	84.7 (1.4)
		(800, 20)	7.6 (2.7)	78.2 (2.9)	39.4 (1.5)	84.6 (1.9)	90.7 (1.2)	87.3 (0.9)
2	-0.5	(400, 10)	13.7 (4.1)	73.9 (3.9)	43.0 (2.9)	15.6 (3.9)	72.0 (3.7)	43.1 (2.8)
		(800, 20)	12.5 (3.0)	76.2 (2.3)	42.3 (2.3)	19.3 (2.8)	76.4 (3.2)	48.9 (3.0)
	0	(400, 10)	1.3 (3.9)	74.3 (3.5)	42.0 (2.7)	13.1 (4.1)	72.5 (3.2)	42.0 (2.7)
		(800, 20)	9.2 (2.4)	77.7 (2.9)	40.6 (1.9)	17.6 (3.3)	77.2 (2.4)	47.2 (2.8)
	0.5	(400, 10)	10.0 (4.8)	74.6 (4.0)	41.4 (2.8)	11.9 (4.2)	72.4 (3.7)	41.3 (2.8)
		(800, 20)	7.2 (3.8)	78.1 (2.7)	39.8 (2.1)	16.5 (3.2)	76.6 (2.1)	48.0 (2.5)

Sensitivity, specificity and overall accuracy are given as percentages.
Reported values are means and standard deviations over 500 simulations.

Table S6: Classification performance of proposed classifiers with high-dimensional continuous covariates when hazard models are misspecified.

			$I(\widehat{\xi}_i^{(0)} > 0.5)$			$I(\widehat{\xi}_i^{(1)} > 0.5)$		
Scenario	τ	$(n,\ J)$	Sensitivity	Specificity	Overall	Sensitivity	Specificity	Overall
1	-0.5	(100, 200)	39.3 (6.6)	62.2 (6.6)	49.9 (5.0)	82.0 (3.5)	75.4 (4.1)	77.1 (3.8)
		(200, 400)	42.7 (3.7)	59.5 (4.2)	49.2 (2.7)	85.5 (3.8)	72.1 (3.8)	79.4 (3.1)
	0	(100, 200)	37.6 (6.9)	61.0 (7.4)	48.4 (5.7)	80.1 (3.1)	73.7 (4.9)	77.0 (3.6)
		(200, 400)	40.1 (4.2)	57.9 (5.1)	47.9 (3.2)	83.2 (2.6)	71.9 (2.7)	78.9 (3.5)
	0.5	(100, 200)	36.5 (6.7)	59.3 (7.4)	47.0 (4.7)	79.8 (3.2)	72.3 (3.6)	75.7 (3.3)
		(200, 400)	39.4 (4.0)	57.1 (5.0)	46.5 (2.1)	81.5 (3.0)	68.7 (2.0)	77.3 (3.2)
2	-0.5	(100, 200)	41.1 (6.8)	62.9 (6.9)	51.2 (4.6)	42.2 (4.2)	66.5 (2.8)	51.6 (4.5)
		(200, 400)	42.3 (3.6)	60.0 (4.6)	49.8 (2.7)	43.4 (3.1)	63.4 (2.6)	56.6 (3.1)
	0	(100, 200)	37.1 (8.1)	60.6 (7.3)	48.2 (5.2)	39.0 (3.4)	61.2 (3.5)	52.0 (4.6)
		(200, 400)	39.9 (4.5)	57.9 (5.3)	47.4 (3.0)	41.3 (2.9)	58.2 (2.9)	53.7 (3.4)
	0.5	(100, 200)	35.5 (6.3)	59.0 (8.4)	46.2 (4.6)	36.5 (4.1)	60.4 (3.6)	50.9 (4.3)
		(200, 400)	37.8 (3.8)	56.2 (6.2)	44.1 (2.6)	37.8 (3.3)	57.3 (2.8)	52.4 (3.0)

Sensitivity, specificity and overall accuracy are given as percentages.
Reported values are means and standard deviations over 500 simulations.

Table S7: Accuracy of coefficient estimates with low-dimensional binary covariates.

			$\widehat{\alpha}$	$\widehat{\boldsymbol{\beta}}$		
Scenario	τ	$(n,\ J)$	Bias	Sensitivity	Specificity	Overall
1	-0.5	(400, 10)	0.48 (0.07)	100 (0)	71.4 (5.1)	80.0 (4.8)
		(800, 20)	0.49 (0.05)	100 (0)	79.6 (4.9)	85.7 (4.7)
	0	(400, 10)	0.48 (0.07)	100 (0)	70.5 (5.5)	79.3 (5.2)
		(800, 20)	0.49 (0.04)	100 (0)	78.7 (5.1)	85.1 (5.0)
	0.5	(400, 10)	0.49 (0.06)	100 (0)	69.9 (6.0)	78.9 (5.7)
		(800, 20)	0.48 (0.05)	99.9 (0.7)	77.8 (5.3)	84.5 (5.2)
2	-0.5	(400, 10)	0.48 (0.07)	100 (0)	73.0 (4.6)	81.1 (4.3)
		(800, 20)	0.50 (0.05)	99.9 (0.7)	80.0 (5.1)	86.0 (5.0)
	0	(400, 10)	0.48 (0.07)	100 (0)	70.3 (5.3)	79.2 (5.0)
		(800, 20)	0.48 (0.05)	100 (0)	78.4 (5.0)	84.9 (4.9)
	0.5	(400, 10)	0.49 (0.06)	99.9 (0.7)	69.8 (5.1)	78.8 (4.8)
		(800, 20)	0.50 (0.05)	100 (0)	77.1 (5.3)	84.0 (5.2)

Sensitivity, specificity and overall accuracy are given as percentages.
Reported values are means and standard deviations over 500 simulations.

Table S8: Accuracy of coefficient estimates with low-dimensional continuous covariates.

			$\widehat{\alpha}$	$\widehat{\boldsymbol{\beta}}$		
Scenario	τ	$(n,\ J)$	Bias	Sensitivity	Specificity	Overall
1	-0.5	(400, 10)	0.44 (0.03)	100 (0)	70.9 (5.4)	79.6 (5.2)
		(800, 20)	0.48 (0.02)	100 (0)	75.0 (5.0)	82.5 (4.9)
	0	(400, 10)	0.45 (0.03)	100 (0)	70.8 (5.3)	79.6 (5.0)
		(800, 20)	0.47 (0.02)	100 (0)	73.6 (4.5)	81.5 (4.4)
	0.5	(400, 10)	0.44 (0.03)	99.9 (0.2)	69.5 (5.1)	78.7 (4.8)
		(800, 20)	0.47 (0.02)	100 (0)	73.8 (4.5)	81.7 (4.4)
2	-0.5	(400, 10)	0.43 (0.02)	100 (0)	70.3 (5.3)	79.2 (6)
		(800, 20)	0.46 (0.01)	100 (0)	74.7 (4.6)	82.3 (4.5)
	0	(400, 10)	0.45 (0.02)	99.9 (0.2)	69.5 (5.0)	78.7 (4.8)
		(800, 20)	0.47 (0.01)	100 (0)	73.3 (4.7)	81.3 (4.6)
	0.5	(400, 10)	0.45 (0.02)	100 (0)	67.8 (4.4)	77.5 (4.6)
		(800, 20)	0.47 (0.01)	99.9 (0.3)	72.9 (4.6)	81.0 (4.5)

Sensitivity, specificity and overall accuracy are given as percentages.
Reported values are means and standard deviations over 500 simulations.

Figure S1: Time to First Recurrence Infection for 23 subjects with recurrence infections.

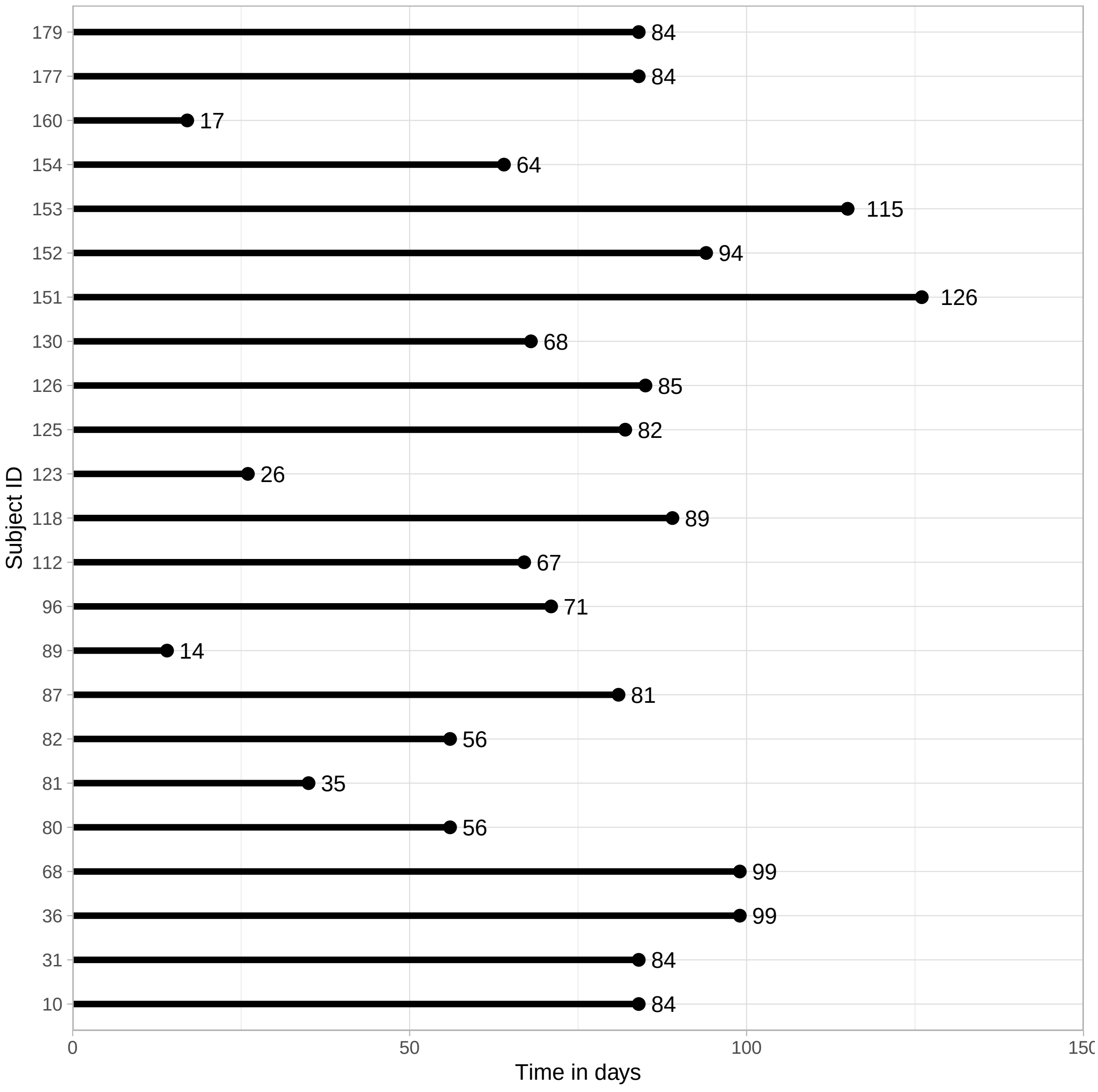

Figure S2: The BIC curve with different values of the tuning parameter ν. The BIC attains its minimum at $\nu = 2.05$.

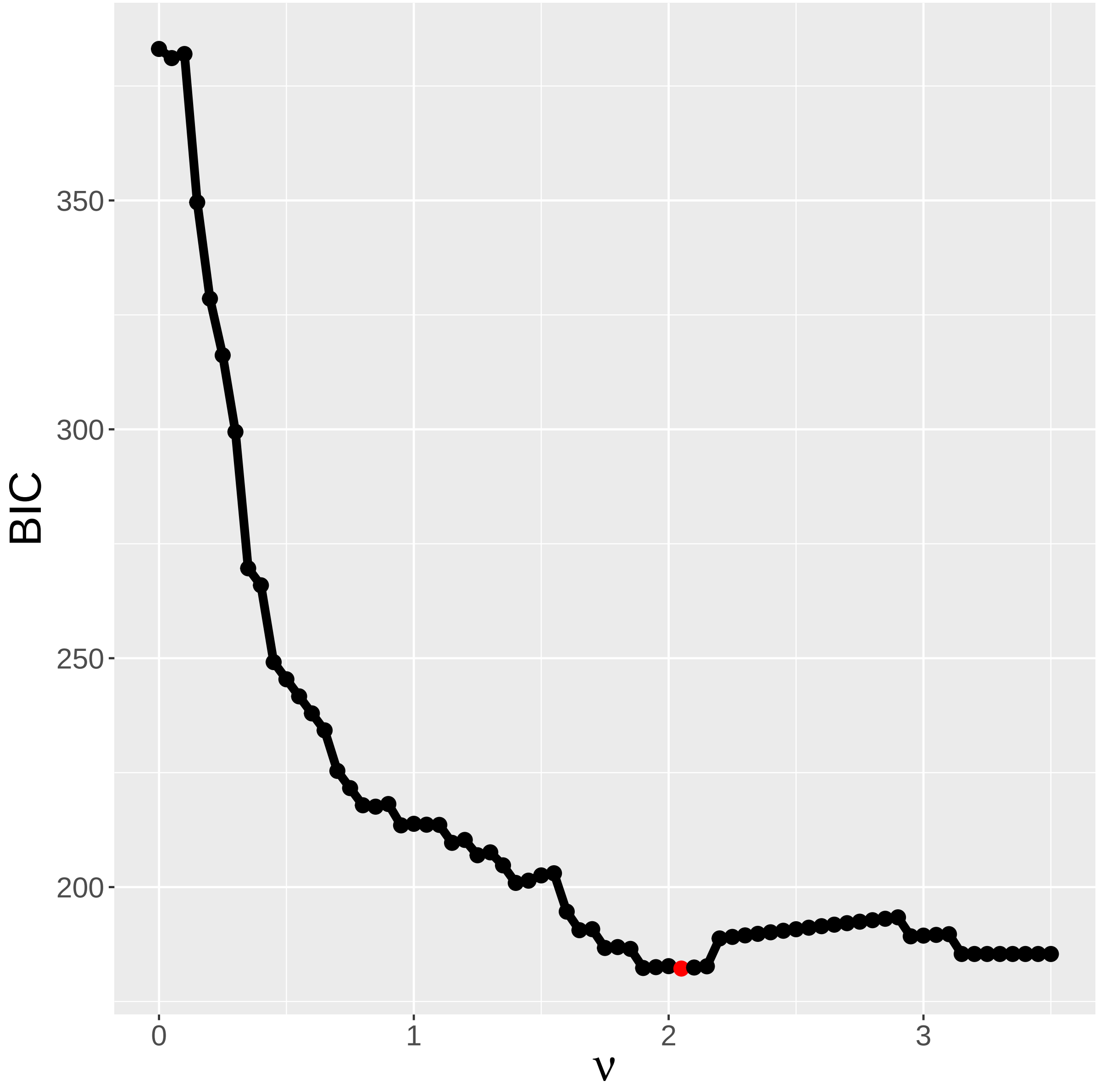

Figure S3: Goodness-of-fit model diagnosis for the *P. vivax* malaria data using $\nu = 0.8$

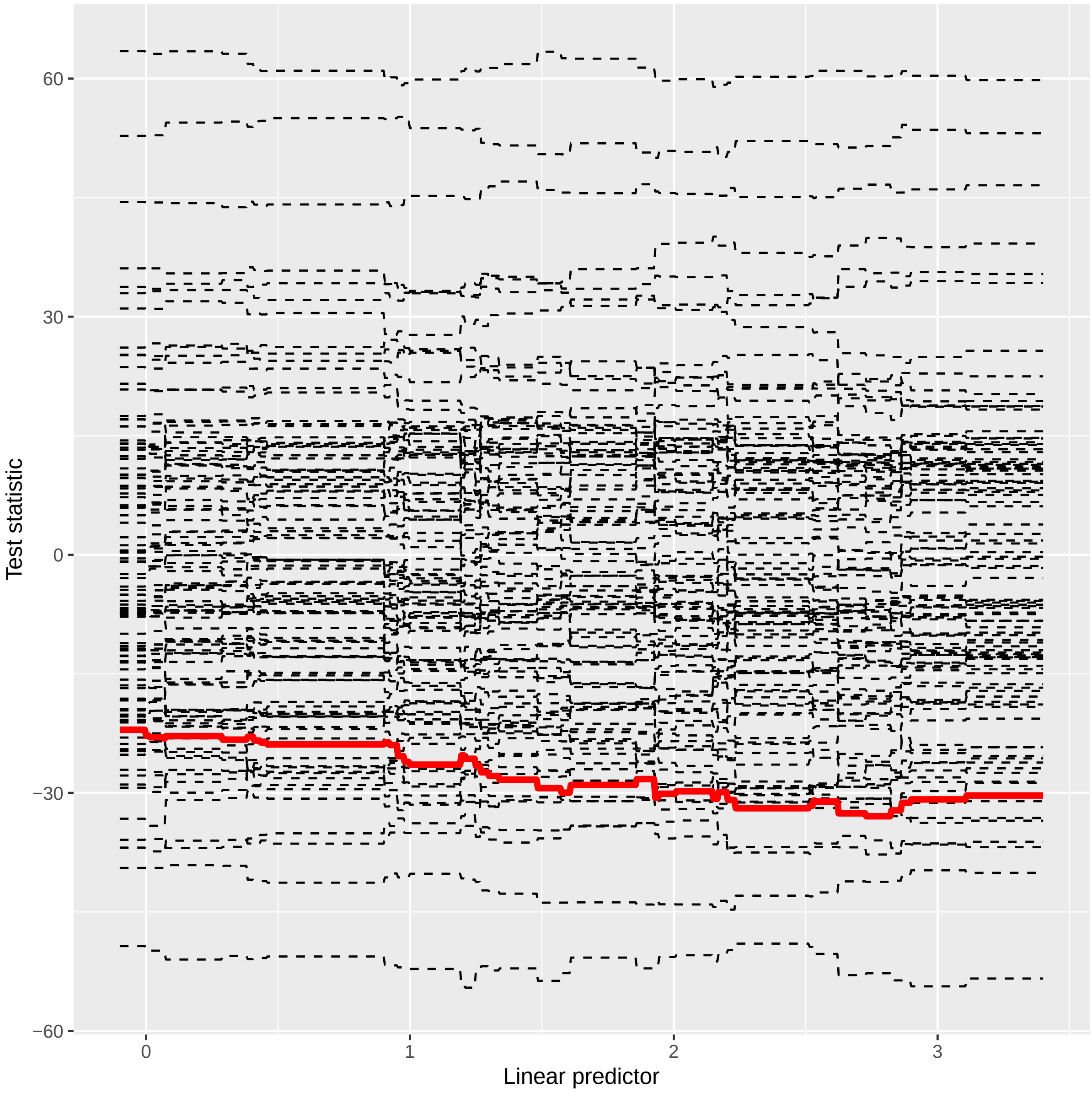

Table S9: Classification of the first recurrent infection based on our proposed method ($\nu = 0.8$).

Recurrence Pair	Days to Recurrence	Baseline Variants	$\widehat{\boldsymbol{\beta}}$	$\widehat{\xi}^{(0)}$	Recurrence Variants	Variant Prevalence	$\widehat{\xi}^{(1)}$	Class by our method	Class by Lin et al.
10 $\rightarrow$ 10R	84	CAM.00	1.194	0.793	CAM.00	0.590	0.996	Relapse	Relapse
		CAM.01	0		CAM.11	0.077			
					CAM.15	0.013			
31 $\rightarrow$ 31R	84	CAM.00	1.194	0.935	CAM.16	0.006	0.992	Relapse	Relapse
		CAM.02	0.075						
		CAM.04	1.245						
		CAM.31	0						
36 $\rightarrow$ 36R	99	CAM.00	1.194	0.897	CAM.01	0.269	0.628	Relapse	Relapse
		CAM.01	0		CAM.02	0.41			
		CAM.02	0.075		CAM.07	0.192			
		CAM.03	-0.293		CAM.17	0.064			
		CAM.04	1.245						
		CAM.05	-0.274						
		CAM.06	-0.292						
		CAM.07	0.287						
		CAM.09	0.068						
		CAM.11	0						
68 $\rightarrow$ 68R	99	CAM.00	1.194	0.936	CAM.10	0.077	0.998	Relapse	Relapse
		CAM.02	0.075						
		CAM.04	1.245						
		CAM.10	0.022						
80 $\rightarrow$ 80R	56	CAM.00	1.194	0.951	CAM.00	0.590	0.000	Reinfection	Reinfection
		CAM.04	1.245		CAM.01	0.269			
		CAM.05	-0.274		CAM.02	0.410			
		CAM.08	0.384		CAM.03	0.295			
		CAM.09	0.068		CAM.05	0.231			
		CAM.24	0.207		CAM.06	0.231			
		CAM.27	0		CAM.07	0.192			

(Continued on next page)

Table S9 (continued from previous page)

Recurrence Pair	Days to Recurrence	Baseline Variants	$\widehat{\boldsymbol{\beta}}$	$\widehat{\xi}^{(0)}$	Recurrence Variants	Variant Prevalence	$\widehat{\xi}^{(1)}$	Class by our method	Class by Lin et al.
					CAM.08	0.154			
					CAM.12	0.064			
					CAM.41	0.013			
81 → 81R	35	CAM.00	1.194	0.793	CAM.00	0.590	0.975	Relapse	Relapse
		CAM.01	0		CAM.01	0.269			
		CAM.51	0						
82 → 82R	56	CAM.00	1.194	0.910	CAM.00	0.590	0.670	Relapse	Relapse
		CAM.03	-0.293		CAM.01	0.269			
		CAM.04	1.245		CAM.03	0.295			
		CAM.10	0.022		CAM.46	0.006			
87 → 87R	81	CAM.00	1.194	0.882	CAM.00	0.590	0.616	Relapse	Relapse
		CAM.01	0		CAM.07	0.192			
		CAM.02	0.075		CAM.08	0.154			
		CAM.08	0.384		CAM.53	0.013			
		CAM.24	0.207						
89 → 89R	14	CAM.00	1.194	0.953	CAM.01	0.269	0.109	Reinfection	Reinfection
		CAM.04	1.245		CAM.09	0.077			
		CAM.06	-0.292		CAM.20	0.026			
		CAM.08	0.384		CAM.27	0.038			
		CAM.10	0.022						
		CAM.12	0.307						
96 → 96R	71	CAM.00	1.194	0.955	CAM.00	0.590	0.992	Relapse	Relapse
		CAM.02	0.075		CAM.30	0.013			
		CAM.04	1.245						
		CAM.08	0.384						
112 → 112R	67	CAM.00	1.194	0.963	CAM.00	0.590	0.847	Relapse	Relapse
		CAM.01	0		CAM.01	0.269			
		CAM.02	0.075		CAM.02	0.410			

(Continued on next page)

Table S9 (continued from previous page)

Recurrence Pair	Days to Recurrence	Baseline Variants	$\widehat{\boldsymbol{\beta}}$	$\widehat{\xi}^{(0)}$	Recurrence Variants	Variant Prevalence	$\widehat{\xi}^{(1)}$	Class by our method	Class by Lin et al.
		CAM.04	1.245						
		CAM.07	0.287						
		CAM.12	0.307						
		CAM.40	0						
		CAM.42	0						
		CAM.60	0						
118 → 118R	89	CAM.08	0.384	0.631	CAM.01	0.269	0.012	Reinfection	Reinfection
					CAM.02	0.410			
					CAM.25	0.006			
					CAM.39	0.006			
123 → 123R	26	CAM.00	1.194	0.805	CAM.00	0.590	0.720	Relapse	Reinfection
		CAM.02	0.075		CAM.01	0.269			
125 → 125R	82	CAM.02	0.075	0.556	CAM.00	0.590	0.000	Reinfection	Reinfection
					CAM.01	0.269			
					CAM.02	0.410			
					CAM.04	0.346			
					CAM.09	0.077			
					CAM.13	0.006			
					CAM.14	0.026			
					CAM.38	0.006			
					CAM.45	0.006			
126 → 126R	85	CAM.00	1.194	0.890	CAM.01	0.269	0.968	Relapse	Relapse
		CAM.01	0		CAM.07	0.192			
		CAM.02	0.075		CAM.33	0.006			
		CAM.03	-0.293						
		CAM.04	1.245						
		CAM.05	-0.274						
		CAM.06	-0.292						

(Continued on next page)

Table S9 (continued from previous page)

Recurrence Pair	Days to Recurrence	Baseline Variants	$\widehat{\boldsymbol{\beta}}$	$\widehat{\xi}^{(0)}$	Recurrence Variants	Variant Prevalence	$\widehat{\xi}^{(1)}$	Class by our method	Class by Lin et al.
		CAM.07	0.287						
		CAM.22	0						
		CAM.50	0						
130 → 130R	68	CAM.00	1.194	0.936	CAM.00	0.590	0.997	Relapse	Relapse
		CAM.02	0.075		CAM.04	0.346			
		CAM.03	-0.293		CAM.12	0.064			
		CAM.04	1.245						
		CAM.12	0.307						
151 → 151R	126	CAM.03	-0.293	0.492	CAM.00	0.590	0.242	Reinfection	Reinfection
		CAM.05	-0.274		CAM.08	0.154			
		CAM.08	0.384		CAM.14	0.026			
					CAM.64	0.006			
152 → 152R	94	CAM.00	1.194	0.793	CAM.00	0.590	0.157	Reinfection	Reinfection
		CAM.01	0		CAM.01	0.269			
					CAM.05	0.231			
					CAM.07	0.192			
153 → 153R	115	CAM.00	1.194	0.947	CAM.02	0.410	0.586	Relapse	Relapse
		CAM.04	1.245		CAM.20	0.026			
		CAM.07	0.287						
		CAM.55	0						
154 → 154R	64	CAM.00	1.194	0.741	CAM.03	0.295	0.098	Reinfection	Reinfection
		CAM.06	-0.292		CAM.05	0.231			
		CAM.57	0		CAM.06	0.231			
160 → 160R	17	CAM.02	0.075	0.853	CAM.00	0.590	0.000	Reinfection	Reinfection
		CAM.04	1.245		CAM.03	0.295			
		CAM.07	0.287		CAM.05	0.231			
					CAM.10	0.077			
					CAM.61	0.006			

(Continued on next page)

Table S9 (continued from previous page)

Recurrence Pair	Days to Recurrence	Baseline Variants	$\widehat{\boldsymbol{\beta}}$	$\widehat{\xi}^{(0)}$	Recurrence Variants	Variant Prevalence	$\widehat{\xi}^{(1)}$	Class by our method	Class by Lin et al.
177 → 177R	84	CAM.00	1.194	0.947	CAM.01	0.269	0.864	Relapse	Relapse
		CAM.04	1.245						
		CAM.07	0.287						
179 → 179R	84	CAM.03	-0.293	0.485	CAM.01	0.269	0.165	Reinfection	Reinfection
		CAM.05	-0.274		CAM.13	0.006			
		CAM.07	0.287						
		CAM.09	0.068						
		CAM.17	0						
		CAM.22	0						

Table S10: Collapsing of original 67 haplotypes to 32 haplotypes.

Original haplotype	Collapse to
CAM.05	CAM.00
CAM.12	CAM.00
CAM.24	CAM.00
CAM.46	CAM.00
CAM.51	CAM.00
CAM.54	CAM.00
CAM.57	CAM.00
CAM.61	CAM.00
CAM.62	CAM.00
CAM.25	CAM.01
CAM.26	CAM.01
CAM.43	CAM.01
CAM.44	CAM.01
CAM.63	CAM.01
CAM.13	CAM.02
CAM.31	CAM.02
CAM.32	CAM.02
CAM.34	CAM.02
CAM.38	CAM.02
CAM.40	CAM.02
CAM.49	CAM.02
CAM.60	CAM.02
CAM.56	CAM.04
CAM.58	CAM.04
CAM.37	CAM.06
CAM.42	CAM.06
CAM.55	CAM.06
CAM.64	CAM.06
CAM.15	CAM.07
CAM.39	CAM.07
CAM.41	CAM.07
CAM.50	CAM.07
CAM.17	CAM.08
CAM.59	CAM.09
CAM.45	CAM.10

Table S11: Classification of first recurrent infection based on our proposed method when using 32 haplotypes ($\nu = 1.6$).

Recurrence Pair	Days to Recurrence	Baseline Variants	$\widehat{\beta}$	$\widehat{\xi}^{(0)}$	Recurrence Variants	Variant Prevalence	$\widehat{\xi}^{(1)}$	Class by our method	Class by Lin et al.
10 → 10R	84	CAM.00	0.862	0.768	CAM.00	0.679	0.927	Relapse	Relapse
		CAM.11	0		CAM.07	0.218			
					CAM.11	0.077			
31 → 31R	84	CAM.00	0.862	0.913	CAM.16	0.006	0.981	Relapse	Relapse
		CAM.02	0						
		CAM.04	1.157						
36 → 36R	99	CAM.00	0.862	0.913	CAM.01	0.321	0.393	Reinfection	Relapse
		CAM.01	0		CAM.02	0.449			
		CAM.02	0		CAM.07	0.218			
		CAM.03	0		CAM.08	0.218			
		CAM.04	1.157						
		CAM.06	0						
		CAM.07	0						
		CAM.09	0						
		CAM.11	0						
68 → 68R	99	CAM.00	0.862	0.913	CAM.10	0.077	0.995	Relapse	Relapse
		CAM.02	0						
		CAM.04	1.157						
		CAM.10	0						
80 → 80R	56	CAM.00	0.862	0.913	CAM.00	0.679	0	Reinfection	Reinfection
		CAM.04	1.157		CAM.01	0.321			
		CAM.08	0		CAM.02	0.449			
		CAM.09	0		CAM.03	0.295			
		CAM.27	0		CAM.06	0.269			
					CAM.07	0.218			
					CAM.08	0.218			
81 → 81R	35	CAM.00	0.862	0.768	CAM.00	0.679	0.942	Relapse	Relapse

(Continued on next page)

Table S11 (continued from previous page)

Recurrence Pair	Days to Recurrence	Baseline Variants	$\widehat{\beta}$	$\widehat{\xi}^{(0)}$	Recurrence Variants	Variant Prevalence	$\widehat{\xi}^{(1)}$	Class by our method	Class by Lin et al.
		CAM.01	0		CAM.01	0.321			
82 → 82R	56	CAM.00	0.862	0.913	CAM.00	0.679	0.723	Relapse	Relapse
		CAM.03	0		CAM.01	0.321			
		CAM.04	1.157		CAM.03	0.295			
		CAM.10	0						
87 → 87R	81	CAM.00	0.862	0.768	CAM.00	0.679	0.461	Reinfection	Relapse
		CAM.01	0		CAM.07	0.218			
		CAM.02	0		CAM.08	0.218			
		CAM.08	0		CAM.53	0.006			
89 → 89R	14	CAM.00	0.862	0.913	CAM.01	0.321	0.7	Relapse	Reinfection
		CAM.04	1.157		CAM.09	0.077			
		CAM.06	0		CAM.20	0.026			
		CAM.08	0		CAM.27	0.038			
		CAM.10	0						
96 → 96R	71	CAM.00	0.862	0.913	CAM.00	0.679	0.989	Relapse	Relapse
		CAM.02	0		CAM.30	0.013			
		CAM.04	1.157						
		CAM.08	0						
112 → 112R	67	CAM.00	0.862	0.913	CAM.00	0.679	0.739	Relapse	Relapse
		CAM.01	0		CAM.01	0.321			
		CAM.02	0		CAM.02	0.449			
		CAM.04	1.157						
		CAM.06	0						
		CAM.07	0						
118 → 118R	89	CAM.08	0	0.583	CAM.01	0.321	0.003	Reinfection	Reinfection
					CAM.02	0.449			
					CAM.07	0.218			
123 → 123R	26	CAM.00	0.862	0.768	CAM.00	0.679	0.702	Relapse	Relapse

(Continued on next page)

Table S11 (continued from previous page)

Recurrence Pair	Days to Recurrence	Baseline Variants	$\widehat{\beta}$	$\widehat{\xi}^{(0)}$	Recurrence Variants	Variant Prevalence	$\widehat{\xi}^{(1)}$	Class by our method	Class by Lin et al.
		CAM.02	0		CAM.01	0.321			
125 → 125R	82	CAM.02	0	0.583	CAM.00	0.679	0.001	Reinfection	Reinfection
					CAM.01	0.321			
					CAM.02	0.449			
					CAM.04	0.359			
					CAM.09	0.077			
					CAM.10	0.077			
					CAM.14	0.026			
126 → 126R	85	CAM.00	0.862	0.913	CAM.01	0.321	0.969	Relapse	Relapse
		CAM.01	0		CAM.07	0.218			
		CAM.02	0		CAM.33	0.006			
		CAM.03	0						
		CAM.04	1.157						
		CAM.06	0						
		CAM.07	0						
		CAM.22	0						
130 → 130R	68	CAM.00	0.862	0.913	CAM.00	0.679	0.98	Relapse	Relapse
		CAM.02	0		CAM.04	0.359			
		CAM.03	0						
		CAM.04	1.157						
151 → 151R	126	CAM.00	0.862	0.768	CAM.00	0.679	0.828	Relapse	Relapse
		CAM.03	0		CAM.06	0.269			
		CAM.08	0		CAM.08	0.218			
					CAM.14	0.026			
152 → 152R	94	CAM.00	0.862	0.768	CAM.00	0.679	0.799	Relapse	Relapse
		CAM.01	0		CAM.01	0.321			
					CAM.07	0.218			
153 → 153R	115	CAM.00	0.862	0.913	CAM.02	0.449	0.76	Relapse	Relapse

(Continued on next page)

Table S11 (continued from previous page)

Recurrence Pair	Days to Recurrence	Baseline Variants	$\widehat{\beta}$	$\widehat{\xi}^{(0)}$	Recurrence Variants	Variant Prevalence	$\widehat{\xi}^{(1)}$	Class by our method	Class by Lin et al.
		CAM.04	1.157		CAM.20	0.026			
		CAM.06	0						
		CAM.07	0						
154 → 154R	64	CAM.00	0.862	0.768	CAM.00	0.679	0.771	Relapse	Relapse
		CAM.06	0		CAM.03	0.295			
					CAM.06	0.269			
160 → 160R	17	CAM.02	0	0.816	CAM.00	0.679	0.009	Reinfection	Reinfection
		CAM.04	1.157		CAM.03	0.295			
		CAM.07	0		CAM.10	0.077			
177 → 177R	84	CAM.00	0.862	0.913	CAM.01	0.321	0.815	Relapse	Relapse
		CAM.04	1.157						
		CAM.07	0						
179 → 179R	84	CAM.00	0.862	0.768	CAM.01	0.321	0.046	Reinfection	Reinfection
		CAM.03	0		CAM.02	0.449			
		CAM.07	0						
		CAM.08	0						
		CAM.09	0						
		CAM.22	0						

References

Lin FC, Li Q, Lin JT (2020). Relapse or reinfection: Classification of malaria infection using transition likelihoods. *Biometrics*, 76(4): 1351–1363.
